# Extracellular histones, a new class of inhibitory molecules of CNS axonal regeneration

**DOI:** 10.1093/braincomms/fcab271

**Published:** 2021-11-13

**Authors:** Mustafa M Siddiq, Sari S Hannila, Yana Zorina, Elena Nikulina, Vera Rabinovich, Jianwei Hou, Rumana Huq, Erica L Richman, Rosa E Tolentino, Jens Hansen, Adam Velenosi, Brian K Kwon, Stella E Tsirka, Ian Maze, Robert Sebra, Kristin G Beaumont, Carlos A Toro, Christopher P Cardozo, Ravi Iyengar, Marie T Filbin

**Affiliations:** 1 Department of Biological Sciences, Hunter College, City University of New York, New York, NY 10065, USA; 2 Department of Pharmacological Sciences and Institute for Systems Biomedicine, Icahn School of Medicine at Mount Sinai, New York, NY 10029, USA; 3 Department of Human Anatomy and Cell Science, University of Manitoba, Winnipeg, Manitoba R3E 0J9, Canada; 4 Gene Editing and Screening Core Facility, Memorial Sloan Kettering Cancer Center, New York, NY 10065, USA; 5 Department of Physiology and Pharmacology, SUNY Downstate Health Science University, Brooklyn, NY 11203, USA; 6 Praxis Spinal Cord Institute, Vancouver, BC, Canada; 7 International Collaboration on Repair Discoveries, University of British Columbia (UBC), Vancouver, BC, Canada; 8 Department of Pharmacological Sciences, Renaissance School of Medicine at Stony Brook University, Stony Brook, NY 11794-8651, USA; 9 Department of Neuroscience and Pharmacological Sciences, Icahn School of Medicine at Mount Sinai, New York, NY 10029, USA; 10 Department of Genetics and Genomic Studies, Icahn School of Medicine at Mount Sinai, New York, NY 10029, USA; 11 Icahn Institute for Data Science and Genomic Technology, Black Family Stem Cell Institute, New York, NY 10029, USA; 12 Sema4, a Mount Sinai Venture, Stamford, CT, USA; 13 National Center for the Medical Consequences of Spinal Cord Injury, James J. Peters VA Medical Center, New York, NY 10468, USA; 14 Department of Medicine, Icahn School of Medicine at Mount Sinai, New York, NY 10029, USA; 15 Department of Rehabilitation Medicine, Icahn School of Medicine at Mount Sinai, New York, NY 10029, USA

**Keywords:** axons, axonal regeneration, inhibitory molecules, CNS, extracellular histones

## Abstract

Axonal regeneration in the mature CNS is limited by extracellular inhibitory factors. Triple knockout mice lacking the major myelin-associated inhibitors do not display spontaneous regeneration after injury, indicating the presence of other inhibitors. Searching for such inhibitors, we have detected elevated levels of histone H3 in human CSF 24 h after spinal cord injury. Following dorsal column lesions in mice and optic nerve crushes in rats, elevated levels of extracellular histone H3 were detected at the injury site. Similar to myelin-associated inhibitors, these extracellular histones induced growth cone collapse and inhibited neurite outgrowth. Histones mediate inhibition through the transcription factor Y-box-binding protein 1 and Toll-like receptor 2, and these effects are independent of the Nogo receptor. Histone-mediated inhibition can be reversed by the addition of activated protein C *in vitro*, and activated protein C treatment promotes axonal regeneration in the crushed optic nerve *in vivo*. These findings identify extracellular histones as a new class of nerve regeneration-inhibiting molecules within the injured CNS.

## Teaser

Siddiq et al. report that elevated levels of histone H3 in human CSF 24 hours after spinal cord injury and that they are inhibitory to neurite outgrowth. Histone-mediated inhibition can be reversed by the addition of activated protein C *in vitro* and *in vivo*, mediated by phosphorylation of YB-1.

## Graphical Abstract

**Graphical Abstract:**
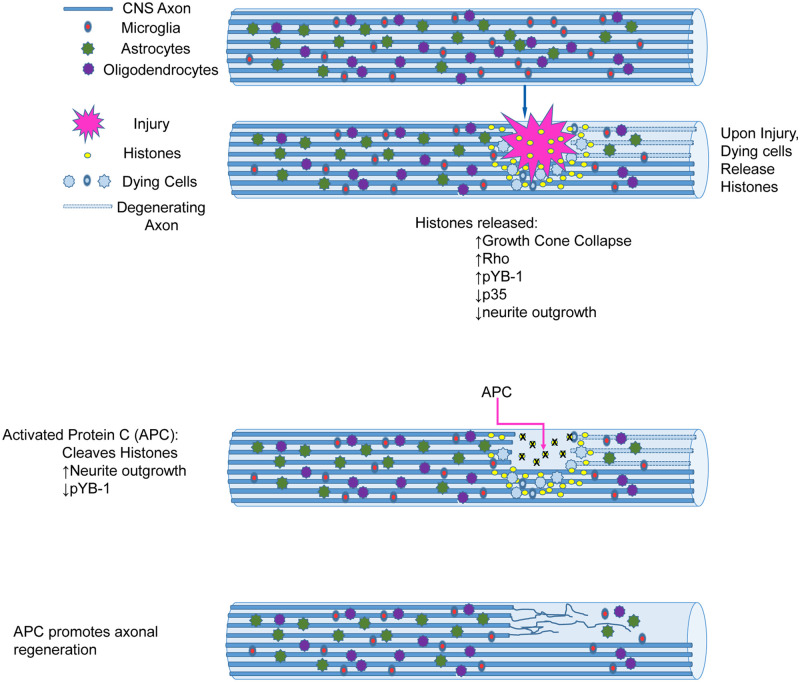

## Introduction

Axonal regeneration following injury is not well understood. Hence the development of therapeutic strategies for neurorepair is challenging. CNS myelin proteins and chondroitin sulphate proteoglycans (CSPGs) are well-characterized inhibitors of axonal growth,^1–4^ but genetic and pharmacological elimination of these factors elicits only a modest increase in axonal regeneration following spinal cord injury (SCI).^5^ This suggests that other inhibitory molecules are present in the CNS environment. Extracellular histones have been detected in neurodegenerative diseases, such as Alzheimer’s disease, scrapie and Parkinson’s disease,^6–8^ and histone H1 has been shown to induce microglial activation and neuronal apoptosis. It has also been reported that histones are released from apoptotic and necrotic cells following cerebral ischaemia, lung injury and myocardial infarction.^7^^,^^9^ Previous studies on sepsis have shown deleterious effects of extracellular histones as they stimulate apoptotic pathways to trigger cell death.^6^ As ischaemia and cell death are key pathophysiological events in axonal injury, we hypothesized histones could be released from damaged cells and contribute to the pathophysiology by inhibiting axonal regeneration. To test our hypothesis, we determined if histone levels are elevated in humans after SCI. We show similar effects after SCI and optic nerve crush in murine models.

Using cell-based assays for axonal regeneration, we show that histones inhibit outgrowth through Toll-like receptor (TLR) 2 and the transcription factor Y-box-binding protein 1 (YB-1). We also show that the protease activated protein C (APC) that cleaves histones^6^ and enhances neuroprotection in cerebral ischaemia^10^ relieves histone induced inhibition of axonal outgrowth. *In vivo* APC promotes regeneration of crushed optic nerves. Histones have been shown to bind receptors such as TLR2 and TLR4, outside the nervous system.^11^ Histones binding to TLR2 and 4 can activate intracellular pathways in those tissues.^12^ In this study, we demonstrate that histones are elevated in response to SCI in humans. Using a rat model, we show that histones inhibit neurite outgrowth *in vitro*, and that these effects are mediated through TLR2 and the transcription factor YB-1. Furthermore, we show that APC promotes regeneration of the rat optic nerve in vivo.

## Materials and methods

### Human CSF fluid from patients with SCI

Human SCI samples and related ASIA scores from fully deidentified patients were obtained from the International Spinal Cord Injury Biobank (ISCIB), which is housed in Vancouver, BC, Canada. Permission for CSF fluid acquisition was granted by the Clinical Research Ethics Board (CREB) of the University of British Columbia, Vancouver, Canada (Ethics certificate of full board approval H19-00690). Samples of fully deidentified CSF from patients with SCI and normal patients were sent to the laboratory of Ravi Iyengar at the Icahn School of Medicine at Mount Sinai (ISMMS), NYC. The IRB review at ISMMS determined IRB approval was not required for these fully deidentified CSF samples, as it is not human research as defined by DHHS and FDA regulations.

All animals are maintained with the highest ethical standards with full IACUC review, and both Hunter College and Icahn School of Medicine have AAALAC accreditation. All animals are given food and water *ad libitum*. They are kept in clean housing conditions with full husbandry. All animals are rested for one week in the vivarium before any surgical procedures. All animals that undergo surgery have full anaesthesia, monitored daily for any pain and discomfort, if so a veterinarian or vet staff are available to check on the welfare of the animal. Pain medication is provided immediately after surgery and for several days post-surgery. Animals in distress or pain are humanely put down with an overdose of isoflurane and monitored until the heart stops beating.

### Rat primary cortical or hippocampal neuron cultures

Rat primary cortical cultures were dissected from postnatal day 1 Sprague Dawley rat brains from both male and female pups. Cortices or hippocampi were incubated twice for 30 min with 0.5 mg/ml papain (Sigma) in plain Neurobasal (NB) media (Invitrogen) with DNase. Papain activity was inhibited by brief incubation in soybean trypsin inhibitor (Sigma). Cell suspensions were layered on an Optiprep density gradient (Sigma) and centrifuged at 1900 × *g* for 15 min at room temperature. The purified neurons were then collected at 1000 × *g* for 5 min and counted. Primary cortical neurons were diluted to 35 000 cells/ml in NB supplemented with B27, L-glutamine and antibiotics, and we seeded 300 µl of the cells suspension to each well and incubated for 24 h. To quantify the outgrowth, we immunostained using a monoclonal antiβIII tubulin antibody (Tuj1; Covance) and Alexa Fluor 568-conjugated anti-mouse IgG (Invitrogen). For quantification, images were taken, and the length of the longest neurite for each neuron was measured using MetaMorph software (Molecular Devices). For Western blots, primary neurons were lysed in radioimmunoprecipitation assay (RIPA) buffer (50 mM Tris-HCl, pH 7.5; 100 mM NaCl; 1% Nonidet P-40; 0.5% deoxycholic acid; 0.1% SDS; 1 mM EDTA) supplemented with protease and phosphatase inhibitors. After determination of protein concentration, the cell lysates were subjected to immunoblot analysis using standard procedures and visualized by enhanced chemiluminescence (ECL). Polyclonal rabbit antibodies directed against phospho-YB-1 and total YB-1 were from Cell Signaling Technology Inc.

### Rat primary dorsal root ganglion neurons

Rat dorsal root ganglion (DRG) neurons extracted from P5- to P7-day-old rat pups from both male and female pups. The neurons were first treated with 0.015% collagenase in Neurobasal-A media for 45 min at 37°C. This was followed by a second incubation in collagenase for 30 min at 37°C, with the addition of 0.1% trypsin and 50 µg/ml DNase I. Trypsin was inactivated with DMEM containing 10% dialyzed foetal bovine serum (FBS), and the ganglia were triturated in Sato’s media.

### Rat primary astrocytes

We prepared mixed cortical cultures from P3–P5 pups from male and female pups. The cortices were removed and the meninges carefully peeled away. They were treated with 0.1% trypsin and 50 µg/ml DNase I for 10 mins at 37°C. Trypsin was inactivated with DMEM containing 10% dialyzed FBS. We triturated the tissue and strained the cell suspension which was seeded into a pre-coated PLL T75 tissue flask in the growth media of DMEM with 10% FBS. Media was replaced within 24 h and then every 3 days until we had a confluent layer of cells (10–14 days). To enrich for astrocytes, the media was replaced with plain NB (no supplements but containing Hepes) and we shaked the culture at 200 RPM at 37°C. The media was aspirated off and we added fresh DMEM with 10% FBS and returned to the incubator overnight. The next day we would trypsinize the remaining cells for 10 min at 37°C, or longer depending on how the cells are lifting off the flask. Trypsin was inactivated with DMEM containing 10% dialyzed FBS, and the astrocytes were counted.

### Neurite outgrowth assay

Monolayers of control or MAG-expressing Chinese hamster ovary (CHO) cells or primary astrocytes were plated on eight well chamber slides as previously described.^13^ Purified P1 hippocampal, P1 cortical, P5–P6 CGN or P5–P6 DRG rat neurons were diluted to 35 000 cells/ml in Sato’s media and treated with either 1 mM dbcAMP (Calbiochem), mixed population or recombinant Histones (1–20 µg/ml) or with APC (1–20 µg/ml). Neurons were incubated for 14–18 h at 37°C and immunostained using a monoclonal anti-βIII tubulin antibody (Tuj1; Covance) and Alexa Fluor 568-conjugated anti-mouse IgG (Invitrogen). For quantification, images were taken, and the length of the longest neurite for each neuron was measured using MetaMorph software (Molecular Devices).

### Microfluidic neurite outgrowth assay

Square microfluidic chambers (150 or 450 microgroove) were purchased from Xona microfluidics. The chambers were sterilized under UV for 15 min and soaked in 70% ethanol for 2 min and allowed to air dry under a sterile TC hood. We used MatTek dishes (P50G-1.5, MatTek corp.) that we pre-coated with PLL overnight, rinsed three times with sterile water and air dried overnight in a sterile hood. Using sterile forceps, we carefully place the microfluidic chamber on the glass area of the MatTek dish, and gently apply pressure to ensure the chambers are sitting on the glass. 3 whole brain P1–2 cortices after papain enzymatic digestion and Optiprep gradient were resuspended in 200 µl of NB with full supplements. We then seeded 15 µl of the neuronal suspension on one side of the microgroove and place in a 37°C incubator for 20 min to allow the neurons to adhere. All wells were filled with 150 µl of supplemented NB. Once we observe neurites growing across the microgroove (2–3 days), we then did treatments on the neurite compartment and wait 48 h before we add 4% Paraformaldehyde (PFA) with 4% Sucrose. We used the Neon transfection (Invitrogen) method to overexpress YB-1.

### Rho activation assay

Rat cortical neurons plated in 10 cm^2^ dishes (approximately 10 million neurons per dish) were used with a commercially available Rho Activation Assay Kit (Millipore). The neurons were placed in plain NB media for 4 h prior to assay. In brief, following the manufacturers protocol, we lysed the cortical neurons on ice with MLB buffer supplemented with anti-protease and anti-phosphatase cocktails (Calbiochem) and spun the lysates at 14 000 × *g* for 5 min. The supernatant was collected and added to 35 µl of agarose beads coupled to Rhotekin Rho Binding Domain and rocked in the cold room for 45 min, a small sample of each supernatant was not added to the beads and was used as total Rho loading controls. Beads were washed three times with MLB buffer and 20 µl 2× Laemmeli buffer was added and samples were prepared for Western blotting. Proteins were separated on pre-cast 4–20% gradient gels (Thermo Fisher Scientific) and transferred to nitrocellulose at 75 V for 1 h. Membranes were successfully probed with rabbit anti-RhoA (1:1000; Cell Signaling Technology) and HRP conjugated anti-rabbit IgG (1:2000; Cell Signaling Technology). Membranes were reacted with Pierce ECL Western Blotting Substrate or SuperSignal West Femto Maximum Sensitivity Substrate (Thermo Fisher Scientific). Densitometric measurements were made using NIH ImageJ software.

### Optic nerve crush experiments

Adult male or female Sprague–Dawley or rats (250–280 g, approximately 8–10 weeks old were anaesthetized with isoflurane and placed in a stereotaxic frame. The right optic nerve was exposed and crushed with fine forceps for 10 s. We placed gelfoam either soaked in PBS or with APC (4.1 mg/ml; Haematologic Technologies, Inc) were placed over the injury site. Three days prior to sacrificing we label the regenerating axons with 5 µl of 1 mg/ml Cholera Toxin B (CTB) coupled to Alexa-488 which we intravitreally inject. Animals were anaesthetized with ketamine (100 mg/kg) and xylazine (20 mg/kg) injected intraperitoneally and transcardial perfusion with 4% PFA after a 14 days postsurgical survival period. When animals were deeply anaesthetized, we transcardially perfused with cold 4% PFA in PBS, pH 7.4. The optic nerves and chiasm attached were dissected out and post-fixed in 4% PFA overnight at 4°C, rinsed for 1 h in PBS and then we prepare them for chemical clearing. Since the advent of the 3DISCO clearing techniques, we are no longer dependent on sectioning the tissue, this method also eliminates bias associated with artefacts produced by sectioning. The whole nerve is placed in a graded series dehydration technique in Tetrahydrofuran (THF; Sigma) diluted in water: 50%, 70%, 80%, 100% and 100% again. Each THF dilution is for 20 min at room temperature on an orbital shaker.^14^ Followed by Dicholoromethane (Sigma) for 5 min at room temperature on an orbital shaker and followed by the clearing agent, Dibenzyl ether (DBE; Sigma) overnight at room temperature on an orbital shaker. Microscope slides are mounted with Fastwell chambers (Electron Microscopy Sciences) and the cleared sample is place on the slide and covered with DBE, a No. 1.5 micro cover glass is used the cover the sample. We image the whole sample on an Olympus Multiphoton microscope with a 25× water immersion lens. All procedures were approved by the IACUC of the Icahn School of Medicine at Mount Sinai in accordance with NIH guidelines. Multiphoton microscopy was performed in the Microscopy CORE at the Icahn School of Medicine at Mount Sinai and was supported with funding from NIH Shared Instrumentation Grant 1S10RR026639-01.

### Multiphoton image analysis

Images were deconvolved using AutoQuant X Software (Media Cybernetics). Deconvolved images were then analysed using Amira Software version 6.0.1 (Thermo Scientific). *Z*-stacks were uploaded to create a 3D volume rendering of the crush site and labelled neurites. For samples containing more than one *Z*-stack, image stacks were aligned and merged using Amira’s merge module. The images were then rotated to position the crush site proximal to the retina on the left with regenerated neurites growing away from the crush site towards the right. The transformed data were resampled and axes swapped such that the XY plane became the cross-section along the length of the nerve. Images were cropped if needed to exclude any partial cross sections of the ends of the nerve caused by image rotation. Labelled regenerated neurites were segmented by thresholding pixels using Amira’s Segmentation Editor. The volume ratio of segmented neurites over total nerve volume was measured for each sample. Study results were then normalized and plotted as a bar graph. The ratio of segmented area over total nerve area for each cross-section was normalized and plotted as area ratio per slice. Area ratio plots were created starting from the behind the crush site proximal to the retina and following along the length of the nerve for the segmented regenerated neurites.

### Cell fractionation and Panomics transcription factor activation arrays

Nuclear and cytosolic extracts were isolated from cortical neurons following instructions from a commercial kit (Pierce).^15^ The supernatant containing the nuclear extract was used in a Panomics Combo Protein-DNA Array (Affymetrix, MA1215) to study transcription factor activation following the manufacturer’s protocol. The array was visualized by enhanced chemiluminescence and determined where we had a positive signal.

### siRNA transfection

All siRNA were purchased from Accell smartpool siRNA (Thermo Scientific), following the manufacturers protocol. The following siRNA for rat were utilized: Rtn4r [Nogo receptor (NgR), EntrezGene 113912], toll-like receptor 2 (EntrezGene 310553) and toll-like receptor 4 (EntrezGene 29260), scrambled non-targeting siRNA was also utilized for controls. Cortical neurons were plated on one side of the microfluidic chambers. We waited 2–4 h after plating to allow the cells to adhere. Utilizing 1 µM siRNA in 150 µl of Accell Delivery Media was added to the neuronal cell bodies and incubated overnight at 37°C incubator. The next morning, we added 150 µl of supplemented NB. 48 h after siRNA-delivery, we added our histones to the neurite side of the chambers. We waited 48 h before stopping the experiment with 4% PFA and 4% sucrose. We then stained the chambers with β-III tubulin and quantified the neurite length.

### Statistical analyses

All analyses were performed using GraphPad Prism software, and data are represented as mean ± SEM. Statistical significance was assessed using paired one-tailed Student’s *t-*tests to compare two groups, and one-way ANOVAs with Bonferroni’s *post hoc* tests to compare between three or more groups.

### Bulk sequencing molecular preparation

We used primary rat cortical neurons for mRNA sequencing, total RNA was extracted using Trizol (Thermo Fisher). All samples RNA integrity was checked by Agilent 2100 Bioanalyzer and all samples had RIN value ≥9.^15^ mRNA libraries for RNA sequencing were constructed using the Truseq stranded mRNA kit (Illumina Inc.) which converts the mRNA in a total RNA sample into a library of template molecules of known strand origin. It captures both coding and non-coding RNA which are poly-adenylated. Briefly, poly-T oligos bound to magnetic beads are used to pull down the poly-A containing mRNA molecules from total RNA and unbound contaminants are washed away. The mRNA is then eluted from the beads and fragmented into smaller pieces using divalent cations under elevated temperature to desired insert sizes depending on the original RNA integrity and required sequencing reads configuration. The resulting mRNA fragment inserts are then reverse transcribed to first strand cDNA using reverse transcriptase and random primers. Use of Actinomycin D during first strand synthesis prevents spurious DNA-dependent synthesis and drives an RNA-dependent synthesis. Strand specificity is achieved during second strand cDNA synthesis by replacing dTTP with dUTP in the Second Strand marking mix which includes the DNA Polymerase I and RNase H. Incorporation of dUTP in second strand synthesis quenches the second strand during amplification. 3′ ends are then adenylated to prevent them from ligating to each other during the adapter ligation reaction. Unique dual index adapters (i5 and i7) are then ligated allowing for greater sample indexing diversity and prepares the ds cDNA for hybridization onto a flow cell. The indexed double-stranded cDNA was then enriched with PCR and purified to create the final cDNA library ready for quantification prior to loading on the flow cell for sequencing.

### Data pre-processing prior to bulk sequencing differential expression analysis pipeline

#### RNASeq and downstream analysis

To reduce experimental artefacts caused by read imbalances we downsampled the sequencing reads of each sample to the number of reads that were detected in the sample with the lowest read counts, as described previously.^16^ Downsampled reads were aligned to the rat reference genome ‘rn6’ using the ensemble annotation and STAR 2.5.4b.^17^ Differentially expressed genes (DEGs) were identified with cufflinks 1.3.0^18^ (FDR 5%, minimum log_2_(fold change) = ±log_2_(1.3)). Up- and downregulated genes were subjected to pathway enrichment analysis using Wikipathways and Gene Ontology biological processes, downloaded from the Enrichr website,^19^^,^^20^ as described previously.^21^

### Data availability

The authors confirm that the data supporting the findings of this study are available within the article [and/or] its supplementary material.

## Results

### Detection of histones in the CSF of humans with SCI

**Figure 1:**
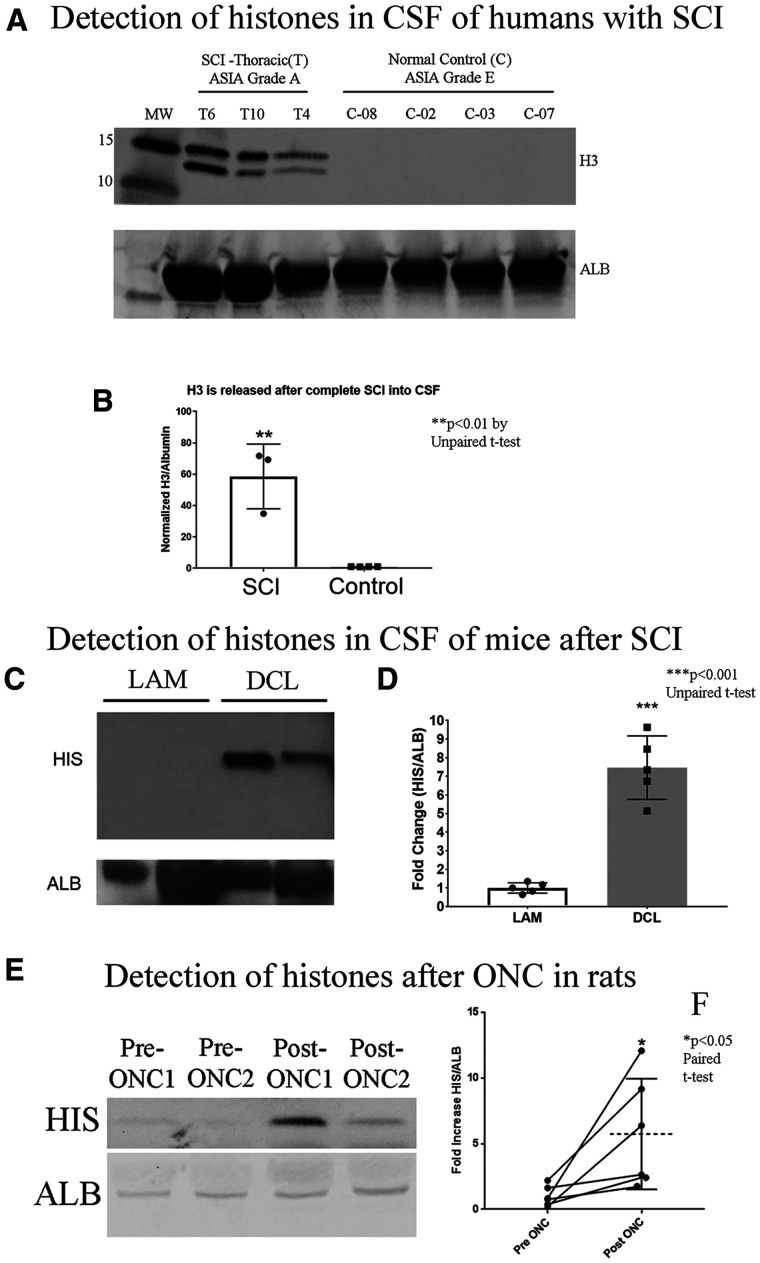
**Elevated levels of extracellular histones detected in the injured CNS.** (**A**) From human CSF collected from patients approximately 24 h post-injury with ASIA score of A (complete impairment, paralysis), and the controls are all normal (ASIA Grade E). On a gradient Western blot, we loaded 30 µgs of sample for each human specimen and probed for histone H3 or Albumin for normalizing control. The H3 is at the predicted size of 17 kDa and we detect it in patients with Thoracic injury (T6, T10 and T4). (**B**) The bar graph is SCI patients comparing to controls, where we see a very significant **P < 0.01 by unpaired *t*-test. (**C**) Elevated levels of Histone H3 (HIS) in the CSF fluid collected from mice with DCL are detected by Western blot, compared to laminectomy (LAM) alone. (**D**) Histone H3 levels are normalized to Albumin (ALB) and in the bar graph is the average of 5 animals (*N* = 5 for both DCL and LAM group) with DCL where we see a significant elevation of free Histone H3 in the CSF fluid. (**E**) Adult rats first had their optic nerves exposed and we applied gelfoam over the uninjured nerve for 48 h (Pre-ONC 1&2), subsequently removing the gelfoam into PBS with protease inhibitor cocktail, we crushed the optic nerve and applied a fresh piece of gelfoam over the injury site. We removed the gelfoam 48 h later (Post-ONC 1&2). We found within the same animal a significant increase in HIS levels after crushing the optic nerve (Post-ONC) than prior to injury (Pre-ONC), data were graphed in **F** (*N* = 6, see Table 1 for full comparison of Pre- and Post-ONC levels of HIS). All statistics performed by unpaired *t*-test, *P < 0.05, **P < 0.01 and ***P < 0.001, except **F**. paired *t*-test.

We obtained CSF from 3 humans with SCI with an ASIA Grade A (complete paralysis), approximately 24 h post-injury from the International Spinal Cord Injury Biobank (ISCIB) who had thoracic (T) injury at levels of T6, T10 and T4. We also obtained samples from 4 control subjects (ASIA Grade E). We ran the CSF samples on a gradient gel and probed for human H3 histones (Fig. 1A; Non-cropped gel Supplementary Fig. 14); the individual running the gel was blinded to what the samples were. We detected histones in the CSF of the human specimens with SCI. None of the control normal human CSF had detectable levels of H3. Elevated Histone levels were observed in samples from subjects with SCI after normalizing band intensities to albumin in the CSF (Fig. 1B; Non-cropped Albumin gel Supplementary Fig. 15).

To validate this finding in the human specimens with Thoracic (T) injury, we performed T8-9 level dorsal column lesions (DCL) in 9–12 weeks old 129S1/SvImJ mice (The Jackson Laboratory) and collected CSF from the injury site 24 h later (*N* = 5, 3 males and 2 females). When compared to a sham group that received laminectomy (LAM) alone, histone levels were significantly higher in CSF obtained from spinal cord-injured animals (Fig. 1C and D; Full gels Supplementary Figs. 16 and 17). To provide further evidence of histone release after CNS injury, we exposed the optic nerve in adult rats and placed gelfoam on the uninjured nerve. These pre-injury gelfoam samples were extracted 48 h later. The nerve was then re-exposed and crushed and a new piece of gelfoam was placed at the injury site for 48 h, enabling us to compare histone levels pre-and post-injury in the same animal. Western blot analysis revealed that histone levels were visibly increased in the optic nerves of individual animals following injury, and overall, this increase was statistically significant (Fig. 1E and F; Full gels Supplementary Figs. 18 and 19). Quantification of the histones released into the gelfoam confirmed that histone levels within individual animals (*N* = 6, 3 males and 3 females) are significantly higher following optic nerve crush (ONC; Table 1).

**Table 1:** Elevated levels of histones-released into gelfoam after ONC in adult rats

Animal#	Pre-ONC	Post-ONC	Fold increase
1	0.21183	6.385752	30.15
2	0.827125	12.08353	14.61
3	0.75167	1.755859	2.34
4	0.384941	2.419942	6.29
5	2.198021	9.15946	4.17
6	1.626405	2.637283	1.62

Below we show the band intensity detected by Western blotting for Histone H3 from the gelfoam placed before (Pre-ONC) and after optic nerve crush (Post-ONC), in the same animal. Average fold increase was 5.74, Paired *t*-test analysis was significant with *P*-value = 0.037.

### mRNA-seq analysis

**Figure 2:**
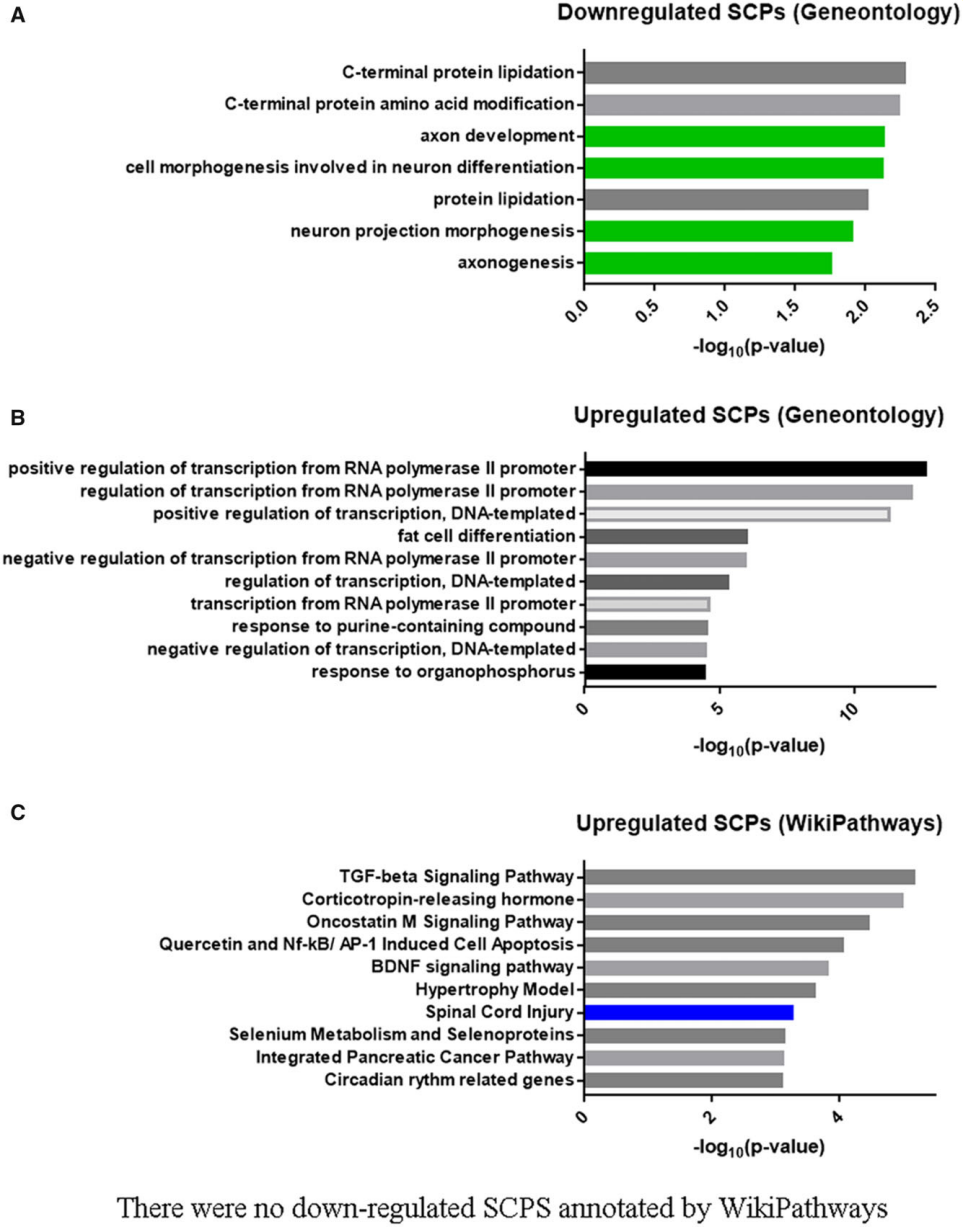
**Bulk mRNA-seq analysis with cortical neurons treated with histones.** (**A**) Up- and Down-regulated genes for histone treated cortical neurons were subjected to pathway enrichment analysis to identify top-ranked pathways. From Geneotology (GO), the top-ranked (according to *P*-values) down-regulated SCPs related to histone treatment of cortical neurons and (**B**) Up-regulated SCPs annotated by GO. (**C**) From Wikipathways, the top-ranked (according to *P*-values) up-regulated SCPs related to histone treatment of cortical neurons, there were no down-regulated SCPs related to histone treatment annotated by Wikipathways.

We also performed bulk mRNA-Seq experiments comparing control (non-treated) primary rat cortical neurons to neurons treated for 2 hrs with a mixed population of histones isolated from calf thymus [10 μg/ml]. The full list of DEGs is found in Table 2. Analysis of the subcellular pathways (SCPs) using WikiPathways revealed that one of the top 10 up-regulated SCPs was for SCI and 4 out of the 7 down-regulated pathways found by Geneontology (GO) involve axon development (Fig. 2A–C). Thus, the mRNA-Seq data support the role of histones in mediating inhibition of neurite outgrowth.

**Table 2:** Full list of Differential Expressed Genes (DEGs) with extracellular histones in rat cortical neurons

NCBI official	NCBI gene description	Log_2_ (fold change)
Gene symbol		
FOSB	FosB proto-oncogene, AP-1 transcription factor subunit	8.825592995
FOS	Fos proto-oncogene, AP-1 transcription factor subunit	8.001262665
NR4A1	nuclear receptor subfamily 4 group A member 1	7.007151127
EGR2	early growth response 2	6.810060024
NPAS4	neuronal PAS domain protein 4	6.753095627
EGR4	early growth response 4	6.658241272
NFATC2	nuclear factor of activated T cells 2	6.276611805
JUNB	JunB proto-oncogene, AP-1 transcription factor subunit	5.752178192
EGR1	early growth response 1	5.627583027
EMP1	epithelial membrane protein 1	5.609884262
VGF	VGF nerve growth factor inducible	5.440786362
MAFF	MAF bZIP transcription factor F	5.31321764
CSRNP1	cysteine and serine rich nuclear protein 1	5.261291504
ATF3	activating transcription factor 3	5.232566833
NR4A2	nuclear receptor subfamily 4 group A member 2	5.082359791
MLF1	myeloid leukaemia factor 1	4.982377529
ARC	activity regulated cytoskeleton associated protein	4.968296051
ARID5A	AT-rich interaction domain 5A	4.937623024
EGR3	early growth response 3	4.929823875
SCG2	secretogranin II	4.620676041
PMAIP1	phorbol-12-myristate-13-acetate-induced protein 1	4.594678402
KLF4	Kruppel like factor 4	4.545769215
JUN	Jun proto-oncogene, AP-1 transcription factor subunit	4.519405842
REM2	RRAD and GEM like GTPase 2	4.510681152
MSANTD1	Myb/SANT DNA binding domain containing 1	4.319052696
SERTAD1	SERTA domain containing 1	4.275292397
GADD45G	growth arrest and DNA damage inducible gamma	4.171737194
CREM	cAMP responsive element modulator	4.167840004
TRIB1	tribbles pseudokinase 1	4.154619694
LMNA	lamin A/C	4.037704468
CYR61	cysteine rich angiogenic inducer 61	3.9201231
BDNF	brain derived neurotrophic factor	3.847639799
TAP1	transporter 1, ATP binding cassette subfamily B member	3.759281635
PPP1R15A	protein phosphatase 1 regulatory subunit 15A	3.681723118
SIK1	salt inducible kinase 1	3.535537004
NPTX2	neuronal pentraxin 2	3.405934334
KLF10	Kruppel like factor 10	3.185355425
ZDBF2	zinc finger DBF-type containing 2	3.153098822
DUSP1	dual specificity phosphatase 1	3.141144753
NR4A3	nuclear receptor subfamily 4 group A member 3	3.080518723
BTG2	BTG anti-proliferation factor 2	3.063263416
SPATA2L	spermatogenesis associated 2 like	3.007558107
NFKBIA	NFKB inhibitor alpha	2.935761213
BTBD8	BTB domain containing 8	2.876153469
RARA	retinoic acid receptor alpha	2.555268049
USP36	ubiquitin specific peptidase 36	2.147712469

We performed bulk mRNA-Seq comparing control (non-treated) primary rat cortical neurons to neurons treated for 2 h with a mixed population of histones isolated from calf thymus (10 μg/ml). In the table, we list the NCBI official gene symbol, gene description and the fold change for all changes with fold change greater than 2.

**Figure 3:**
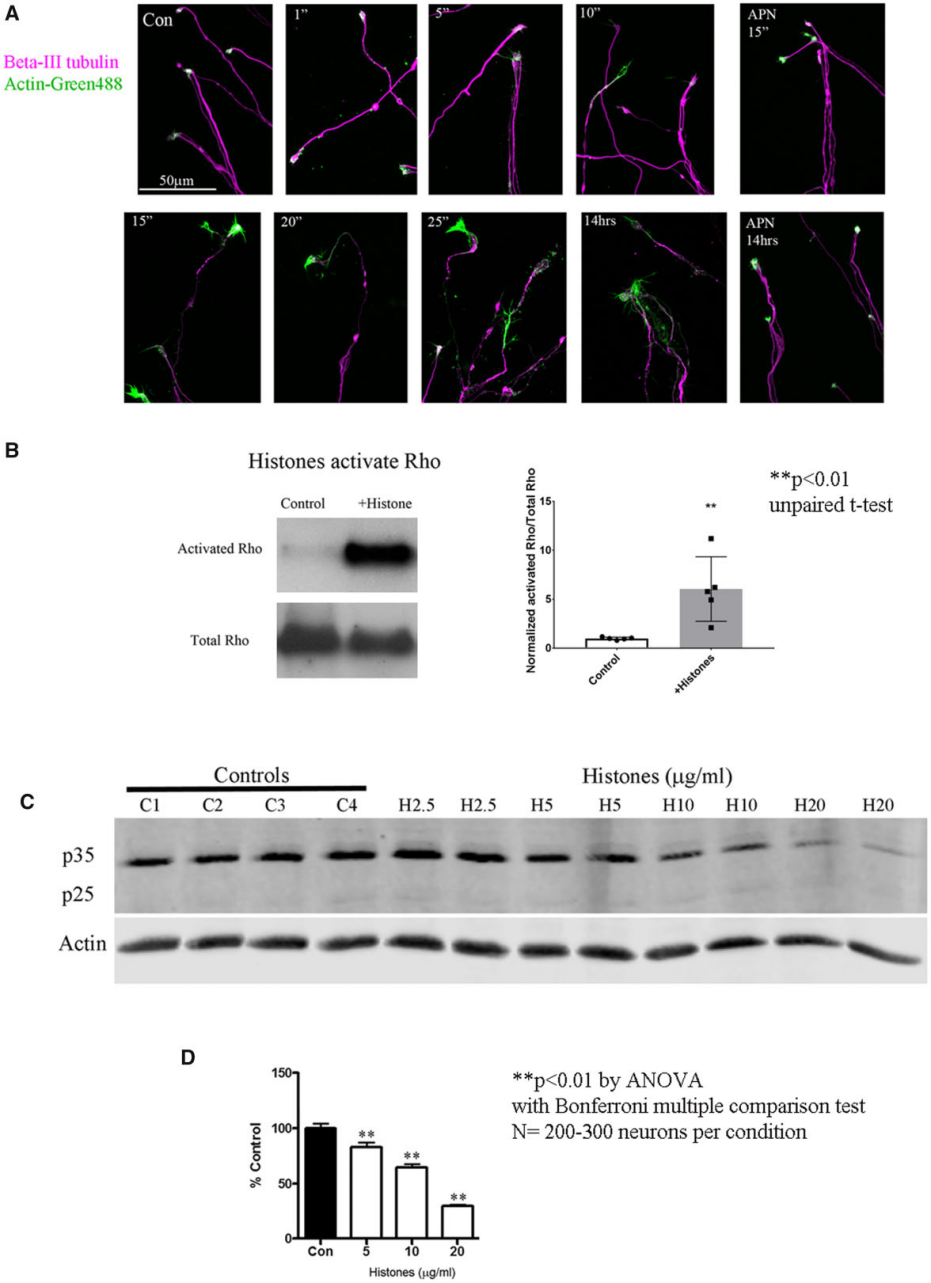
**Extracellular Histones promote growth cone collapse and activate Rho.** (**A**) Cortical neurons on PLL-coated glass plates. Images of β-III tubulin in magenta and actin in green (overlapping magenta and green appear white) displaying growth cones, imaged at 63×. Control, non-treated or Aprotinin (APN) for 15 min or 14 h has no effect on growth cone morphology. Histones applied (1, 5, 10, 15, 20, 25 min or 14 h) results in dystrophic looking bulbs within 15 min. (**B**) Histone applied to cortical neurons induces activation of Rho GTPase, the graph is the average (±SD) with *N* = 5, one sample per condition that was used in 5 different experiments. **P < 0.01 by unpaired *t*-test. (**C**) Histone applied to cortical neurons results in dose-dependent decrease (2.5–20 µg/ml) in p35 levels without any detectable p25. (**D**) Cortical neurons on a permissive CHO monolayer put out long neurites (black bars, Con) but in the presence of histones they put out significantly shorter neurites (white bars). The bar graphs are the average of three independent experiments where we measured *N* = 300 neurons per condition. Statistics for **B** are unpaired *t*-test, for **D** it is one way ANOVA with Bonferroni multiple comparison test, *P < 0.05 and **P < 0.01.

Studies of myelin-associated inhibitors and CSPGs have shown that these molecules inhibit axonal regeneration through activation of the small GTPase RhoA, which mediates growth cone collapse.^22^ To examine the effects of histones on growth cone morphology, we treated cortical neurons with aprotinin (APN) or histones and then immunostained growth cones for β-III tubulin and actin. Cortical neurons treated with aprotinin, a protein of similar size and isoelectric point to histones (but with no structural homology), did not affect growth cones as their morphology was similar to those seen in non-treated cortical neurons (Fig. 3A). By contrast, within 10 min of treatment with histones (10 μg/ml), we observed more actin at the growth cone tips, and this histone effect was very apparent within 15 min (Fig. 3A). Furthermore, the β-III tubulin shows a punctate pattern of staining, suggesting degeneration. By 25 min, dysmorphic end-bulbs were apparent and these were still present at 14 h. With regard to the molecular events underlying these morphological changes, we observed that a 30 min treatment with extracellular histones significantly increased activation of RhoA (Fig. 3B; Non-cropped gels Supplementary Figs. 20 and 21) and also reduced levels of p35 (Fig. 3C; Non-cropped gels Supplementary Figs. 22 and 23), a protein that can overcome MAG-mediated inhibition by activating cyclin-dependent kinase 5.^23^ These observations provide evidence that the signalling pathways activated by CNS myelin, CSPGs and histones converge at key points to mediate inhibition of neurite outgrowth, and importantly, indicate that histones affect cytoskeletal dynamics.

**Figure 4:**
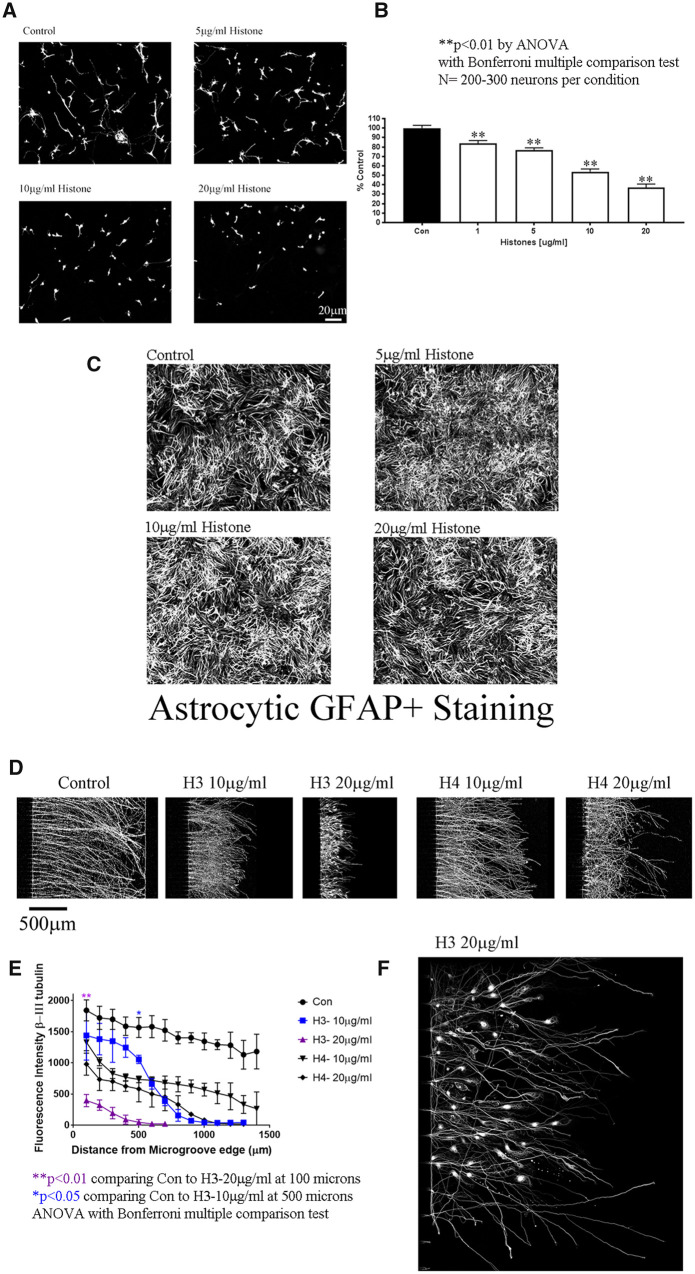
**Extracellular histones are inhibitory to neurite outgrowth.** (**A**) Primary rat cortical neurons on an astrocytic monolayer put out long neurites (Control), but in the presence of increasing concentrations of a mixed population histone preparation they put out dose-dependent shorter neurites as determined by β-III tubulin staining. *N* = 200–300 neurons per condition. (**B**) Primary rat cortical neurons on an astrocytic monolayer put out long neurites (black) as quantified by longest length by Metamorph analysis, but there is a dose-dependent decrease in neurite length in the presence of histones. This bar graph is the average of three independent experiments with a minimum of *N* = 200–300 neurons per group. (**C**) Representative images of GFAP+ astrocytic monolayers in the absence or presence of 5–20 µg/ml as indicated. (**D**) Using PLL-coated microfluidic chambers, cortical neurons grow long neurites across the 450 µM microgroove as detected by β-III tubulin. Treating the neurite growing compartment with either recombinant H3 or H4 Histones resulted in significantly shorter neurites, with H3 having a more potent effect, as seen in close-up image of H3 with 20 µg/ml H3 in **E** and **F**. We quantified the neurite outgrowth in the chambers using Image J, each treatment is the average of three independent experiments. Statisitcs is one-way ANOVA with Bonferroni multiple comparison test. The purple ** at 100 microns is for H3 20 µg/ml and it is significantly shorter compared to controls, and all points subsequent comparing H3 20 µg/ml to controls from 100 to 700 microns were **P < 0.01. The blue * at 500 microns is for H3 10 µg/ml, when it is significantly shorter compared to controls *P < 0.05.

To assess the effects of extracellular histones on axonal growth, we performed a series of neurite outgrowth assays. Using permissive substrates of CHO cells, we observed that histones inhibit neurite outgrowth in a dose-dependent manner for both cortical (Fig. 3D for graph and Supplementary Fig. 1A–C for representative images of cortical neurons on CHO cells) and DRG neurons (Supplementary Fig. 2). We performed a second neurite outgrowth assay using a permissive layer of primary rat astrocytes. Neurite outgrowth was significantly reduced in a dose-dependent manner in response to histone treatment (Fig. 4A and B). This effect was not due to toxicity or the presence of other inhibitory factors, as the astrocyte monolayers were unaffected by the histones (Fig. 4C), and histones did not induce expression of the CSPGs neurocan and brevican in astrocytes (Supplementary Fig. 3). Finally, to elucidate the contributions of specific core histones to inhibition, cortical neurons were plated in microfluidic chambers and recombinant histones H3 and H4 were applied to the neurite compartment. Both histone H3 and H4 inhibited neurite outgrowth, with histone H3 having a more potent effect (Fig. 4D, full chambers are in Supplementary Fig. 5). Quantification of the neurite outgrowth in Fig. 4D is in Fig. 4E. Morphologically, neurites treated with either histone H3 or H4 displayed the prominent end bulbs typical of dystrophic axons (Fig. 4F for H3 and Supplementary Fig. 4).

**Figure 5:**
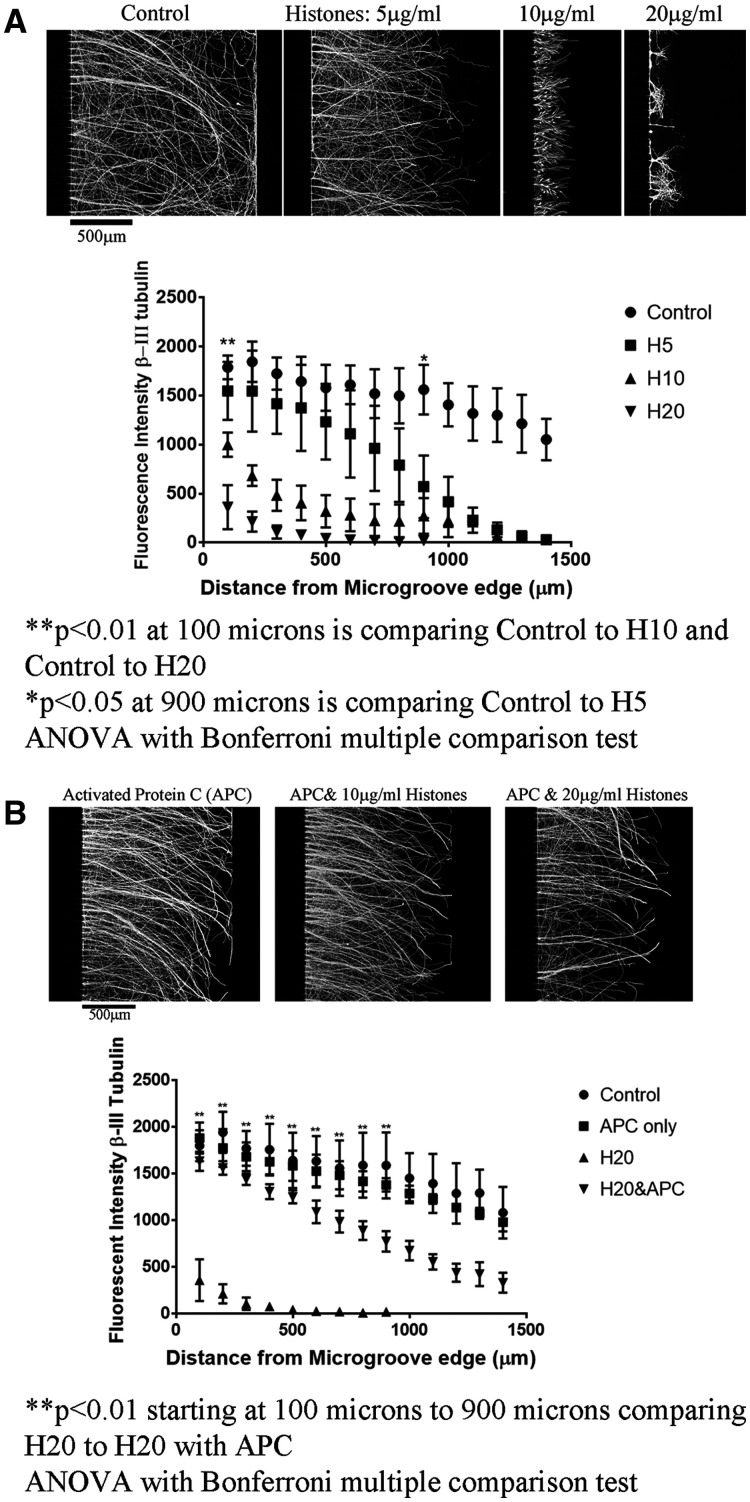
**Activated Protein C (APC) blocks the inhibitory effect of Histones and promotes axonal regeneration after ONC.** (**A**) Cortical neurons plated on PLL coated microfluidic chambers grow robustly across the 450 µm microgroove, determined by β-III tubulin staining. Treating the neurite growing side (right side from the microgroove) only with increasing concentrations of mixed population of histones isolated from calf thymus results in significantly shorter neurites. In the graph we show the average of four independent experiments, we have measurements of β-III tubulin fluorescence in increments of every 100 microns from the microgroove with Control (Aprotinin-treated), H5 (Histones—5 µg/ml), H10 (Histones 10 µg/ml) and H20 (Histones 20 µg/ml). For statistics, the ** at 100 microns is for H10 and H20 being significantly shorter than controls. At 900 microns, H5 is significantly shorter (*) compared to controls. (**B**) APC alone has no effect on neurite outgrowth, combining APC with histones and applying to the neurite side reverses the inhibitory effect of histones, restoring long neurite outgrowth. In the graph we show the average of three independent experiments, we have measurements of β-III tubulin fluorescence in increments of every 100microns from the microgroove with Control (non-treated), APC only (APC-treated), H20 (Histones 20 µg/ml) and H20 (Histones 20 µg/ml with APC). For statistics, the ** is comparing H20 to H20 with APC. Statistics is one-way ANOVA with Bonferroni multiple comparison test, *P < 0.05 and **P < 0.01.

In addition, we used a mixed population of histones isolated from calf thymus and showed a dose-dependent decrease in neurite outgrowth (Fig. 5A, full chambers are in Supplementary Fig. 6). Incubation of cortical neurons with APN has no effect on neurite outgrowth (Fig. 5A). Since APN has no effect on neurite outgrowth, we conclude that the inhibition that is observed is histone-mediated, and not due to non-specific steric hindrance.

Previous studies in other organ systems have reported that the deleterious effects of extracellular histones can be attenuated by activated protein C (APC), a coagulation factor protease that has been shown to cleave histones H3 and H4,^6^ and enhance neuroprotection in cerebral ischaemia.^10^ Using cortical neurons plated in microfluidic chambers, we observed that administration of APC blocked histone-mediated inhibition of neurite outgrowth (Fig. 5B, full chambers are in Supplementary Fig. 7).

**Figure 6:**
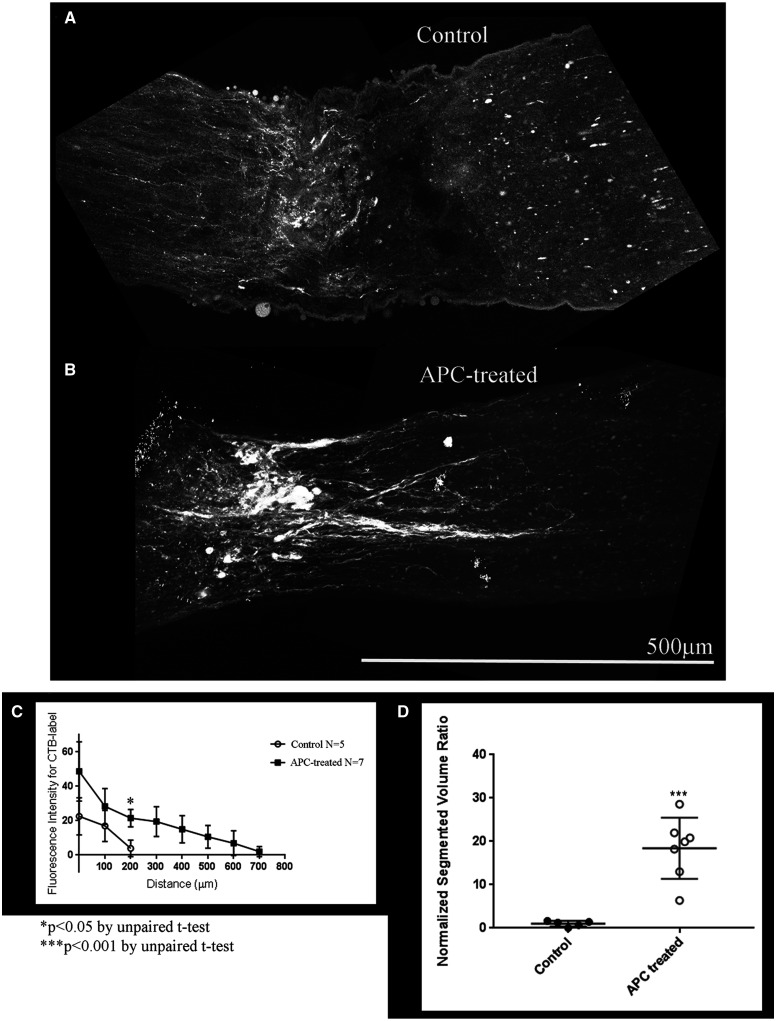
**APC promotes axonal regeneration in the rat ONC model.** (**A**) Using the rat ONC model for axonal regeneration in the CNS, crushed axons without treatment (Control) have CTB labelled axons abruptly halt at the lesion site in this chemically cleared whole nerve. (**B**) Representative optic nerve treated with APC soaked in gelfoam immediately after crushing the axons, the APC-gelfoam is placed over the injury site. In this chemically cleared nerve CTB labelled axons are observed growing through the lesion site. Panels **A** and **B** are approximately 540 optical slices at 0.45 µm increments that is merged together into a two-dimensional represented maximum projection intensity image. Imaged at 25× on an Olympus Multiphoton microscope and scale bar of 500 µm is for **A** and **B**. (**C**) Using ImageJ, we quantitated the axonal regeneration for Control (*n* = 5, white circle) and APC-treated (*n* = 7, black square) every 100 microns from the edge of the crush site. There is a significant difference in the extent of regeneration at 200 microns, and no regenerating fibres were detected past that point in Control animals as determined by unpaired *t*-test, *P < 0.05. (**D**) Using Amira 3D maximum intensity projection of the nerve showing crush site and regenerating neurites labelled with CTB, we determined the Normalized Segmented Volume ratio. Line plot shows ratio of segmented neurite area over total nerve area per cross section along the nerve and we see a significant difference (***, *P* < 0.001 by unpaired *t*-test) with APC-treatment. Each cross-section slice is 497 μm.

**Figure 7:**
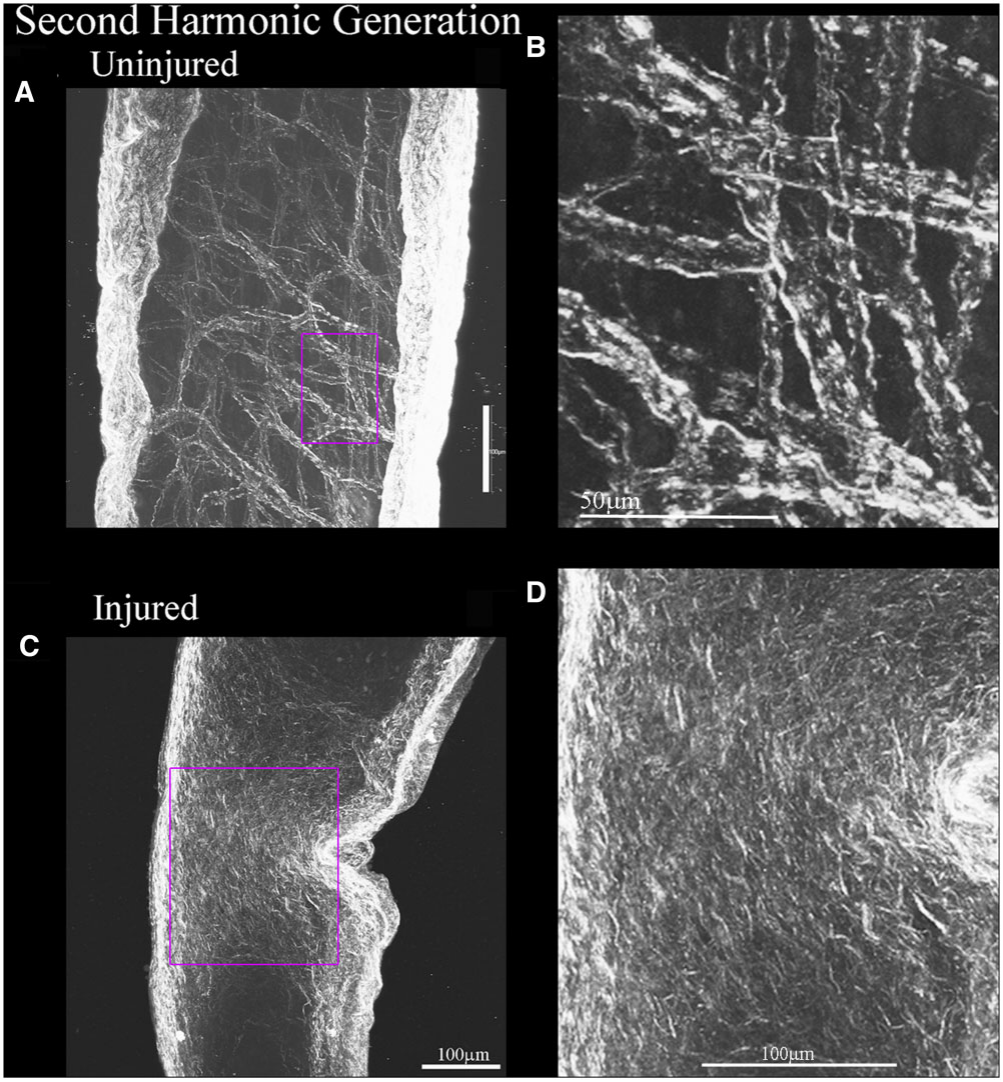
**Second harmonic generation imaging helps reveal the injury site of the crushed optic nerve.** (**A**) Using second harmonic generation imaging on multi-photon microscope, collagen fibres auto-fluoresce. In uninjured whole chemically cleared nerves, we can see the collagen bundles, and the demarcated box in Magenta is where the fibres are shown in enhanced detail in **B**. (**C**) Injured optic nerves display crushed collagen fibres at the injury site, helping to elucidate the injury site and the demarcated box in Magenta displays an enhanced region in **D**. Scale bar is 100 µm in **A**, **C** and **D**, it is 50 μm for **B**.

To determine if APC could promote axonal regeneration *in vivo*, APC-containing gelfoam (*N* = 7, 3 males and 4 females) was applied to the injury site following ONC in rats (Fig. 6A and B). Retinal ganglion cell axons were anterogradely labelled with cholera toxin B-subunit (CTB) at 14 days after injury, and optic nerves were chemically cleared using the 3-DISCO technique.^14^ Crush sites were identified using second harmonic generation, which allows visualization of collagen fibrils (Fig. 7A–D)^24^^,^^25^ and axonal regeneration was quantified by fluorescent intensity from the crush site at 100 µm increments (Fig. 6C) and volumetric analysis of CTB-labelled axons (Fig. 6D and Supplementary Fig. 8). Control animals treated with saline-containing gelfoam (*N* = 5, 2 males and 3 females) displayed limited growth and short CTB-labelled axons crossing the lesion site (Fig. 6A). In APC-treated animals, however, we observed a significant increase in axonal regeneration beyond the site of injury (Fig. 6B and another representative figure with 3D projection of fibres in Supplementary Fig. 9).

**Figure 8:**
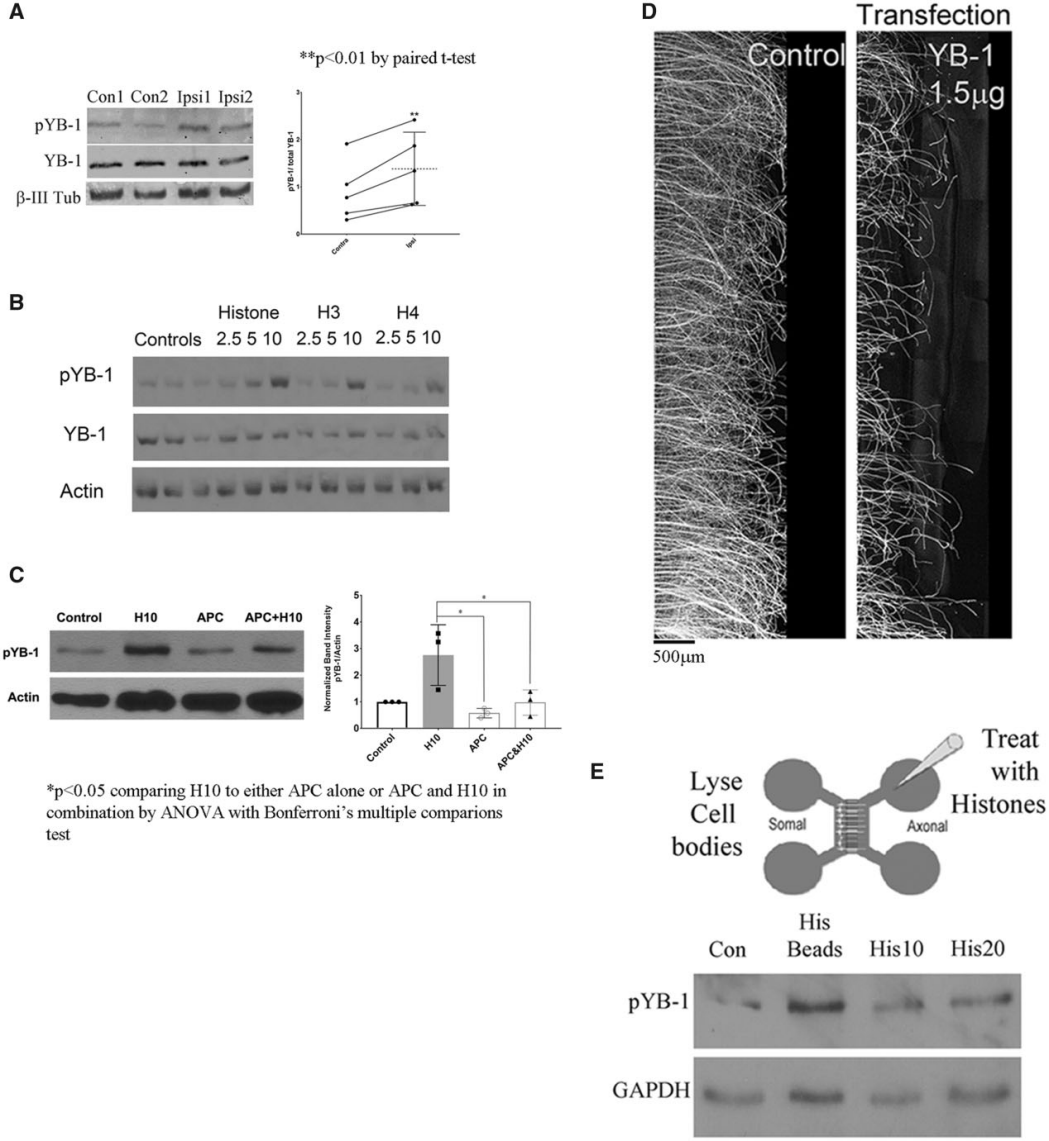
**Histones inhibit neurite outgrowth by an YB-1 mediated mechanism.** (**A**) To confirm if pYB-1 are elevated after injury inducing histone release in the ONC, we crushed the axons of the optic nerve and 48 h later collected the retinal cell layer, ipsilateral side (Ipsi 1&2), and collected the retinal layer of the non-injured, contralateral side (Con1&2) in the same animal and prepared them for Western blots. We found significantly elevated levels of pYB-1 normalized to total YB-1 on the injured (Ipsi) side compared to the non-injured (Contra) side. Statistics is paired *t*-test, **P < 0.01. (**B**) Cortical neurons treated with mixed Histones or recombinant H3 or H4 (2.5, 5 or 10 µg/ml) induce elevation in phosphorylated YB-1 (pYB-1), normalized to YB-1 and Actin levels. (**C**) In cortical neurons treated with 10 μg/ml mixed Histones (H10) we see up-regulation of pYB-1. APC does not significantly alter phosphorylation of YB-1 but it does attenuate Histone-mediated activation of YB-1 compared to Control. The graph is the average of three independent experiments using primary cortical neurons. Statistics is one way ANOVA with Bonferroni multiple comparison test, *P < 0.05. (**D**) Overexpressing the YB-1 plasmid in cortical neurons by NEON transfection and plating in microfluidic chambers results in shorter neurite outgrowth in a dose-dependent manner as determined by β-III-tubulin staining. Scale bar is 500 μm. (**E**) Cartoon of a microfluidic chamber diagrammatically showing where we apply the histones or to the neurites and then lysed the cell bodies 48 h later, on the opposing side. Cortical neurons plated in microfluidic chambers for 4–5 days grow long neurites across the microgroove. Using histones or fluorescent microbeads that are covalently coupled to histones (or non-coupled for controls) and too large to cross the microgrooves, or with 10 or 20 µg/ml mixed Histones (His10 or His20) are applied to the neurite compartment (axonal) only and we wait 48 h before lysing the cell body compartment (somal) with 2XRIPA buffer and run a Western blot. We detect elevation of pYB-1 in the cell bodies normalized to GAPDH.

To elucidate the molecular mechanism underlying histone-mediated inhibition of neurite outgrowth, we treated cortical neurons with histones for 24 h and then plated them onto permissive CHO cell monolayers in the absence of histones.^26^ Neurite outgrowth was severely impaired in these neurons (Supplementary Fig. 10), which suggested that their intrinsic capacity for neuritogenesis had been altered at the molecular level by histones. To determine if this effect involved the activation of transcription factors, we incubated cortical neurons with extracellular histones for 30 or 120 min and analysed the lysates using a Panomics Transcription Factor protein array.^27^ This assay revealed significant increases in eukaryotic YB-1 in histone-treated neurons (Supplementary Fig. 11). To determine if YB-1 was activated in response to injury, we performed optic nerve crushes and collected the ipsilateral and contralateral retinas 48 h later. We observed baseline phosphorylation of YB-1 in the contralateral retina, and this was visibly increased in response to optic nerve crush (Fig. 8A; Non-cropped gels Supplementary Figs. 24–26). We also found that histones H3 and H4, as well as mixed histones, could induce phosphorylation of YB-1 (Fig. 8B, quantification of data is in Supplementary Fig. 12; Non-cropped gels Supplementary Figs. 27–29) and that APC did not affect pYB-1 levels but it did attenuate histone induced activation of YB-1 (Fig. 8C; Non-cropped gels Supplementary Figs. 30 and 31). We also noted that YB-1 expression had a direct impact on neurite outgrowth, as overexpression of YB-1 in cortical neurons resulted in shorter neurites compared to controls (Fig. 8D). As the application of histones to distal neurites is sufficient to inhibit neurite outgrowth (Fig. 4D), and injuring the optic nerve will subsequently lead to elevation of pYB-1 in the retinal ganglion cell layer, it appears that histones may induce phosphorylation of YB-1 through retrograde signalling. To test this hypothesis, the neurite compartments of microfluidic chambers were treated with histones coupled to 6 µm silicone beads, which prevents their diffusion into the somal compartment, while preserving their ability to interact with the neurites. When the histone conjugated beads were applied to the neurites, we observed increased pYB-1 levels in the neuronal cell bodies, confirming a retrograde signal (Fig. 8E; Non-cropped gels Supplementary Figs. 32 and 33).

**Figure 9:**
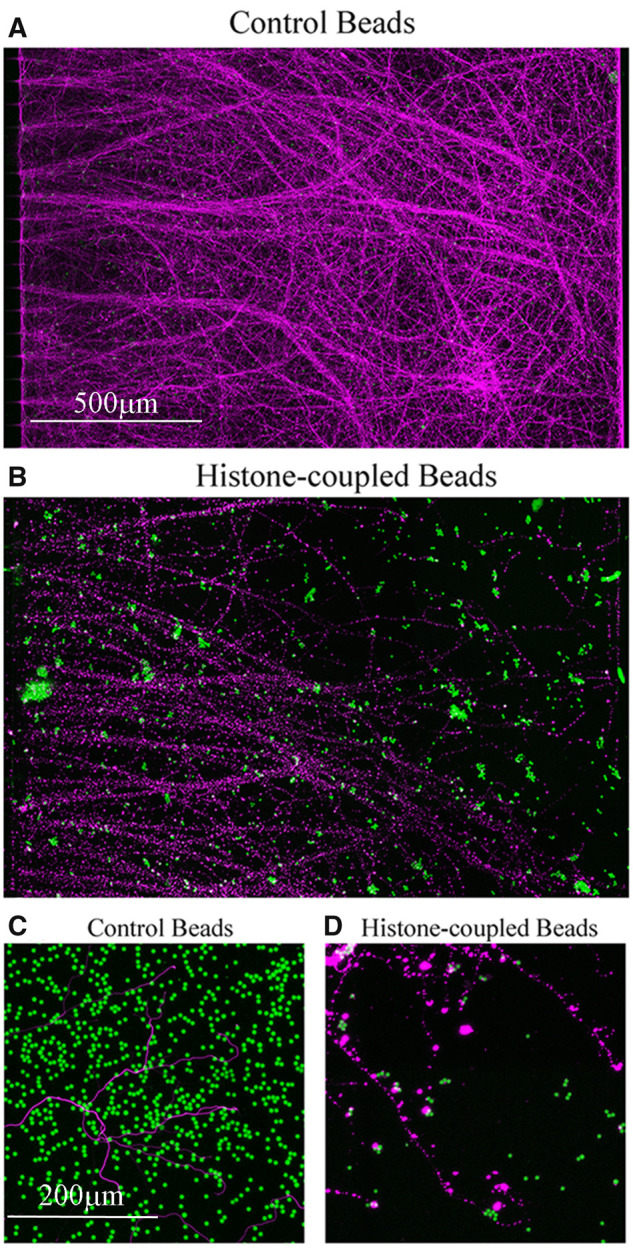
**Immobilized histones coupled to beads inhibited neurite outgrowth.** (**A**) Non-coupled beads (green) had no effect on neurite outgrowth (β-III-tubulin in magenta), (**C**) shows higher magnification. (**B**) Histone-coupled beads (green) appear more aggregated compared to non-coupled beads and significantly inhibited neurite outgrowth. (**D**) Higher magnification of the beaded BIII-tubulin (magenta) with histone-coupled beads. This experiment was reproducibly carried out three times. Micrometer for **A** and **B** is shown in **A**. and it is 500 μm. Micrometer for **C** and **D** is shown in **C**. and it is 200 μm.

**Figure 10:**
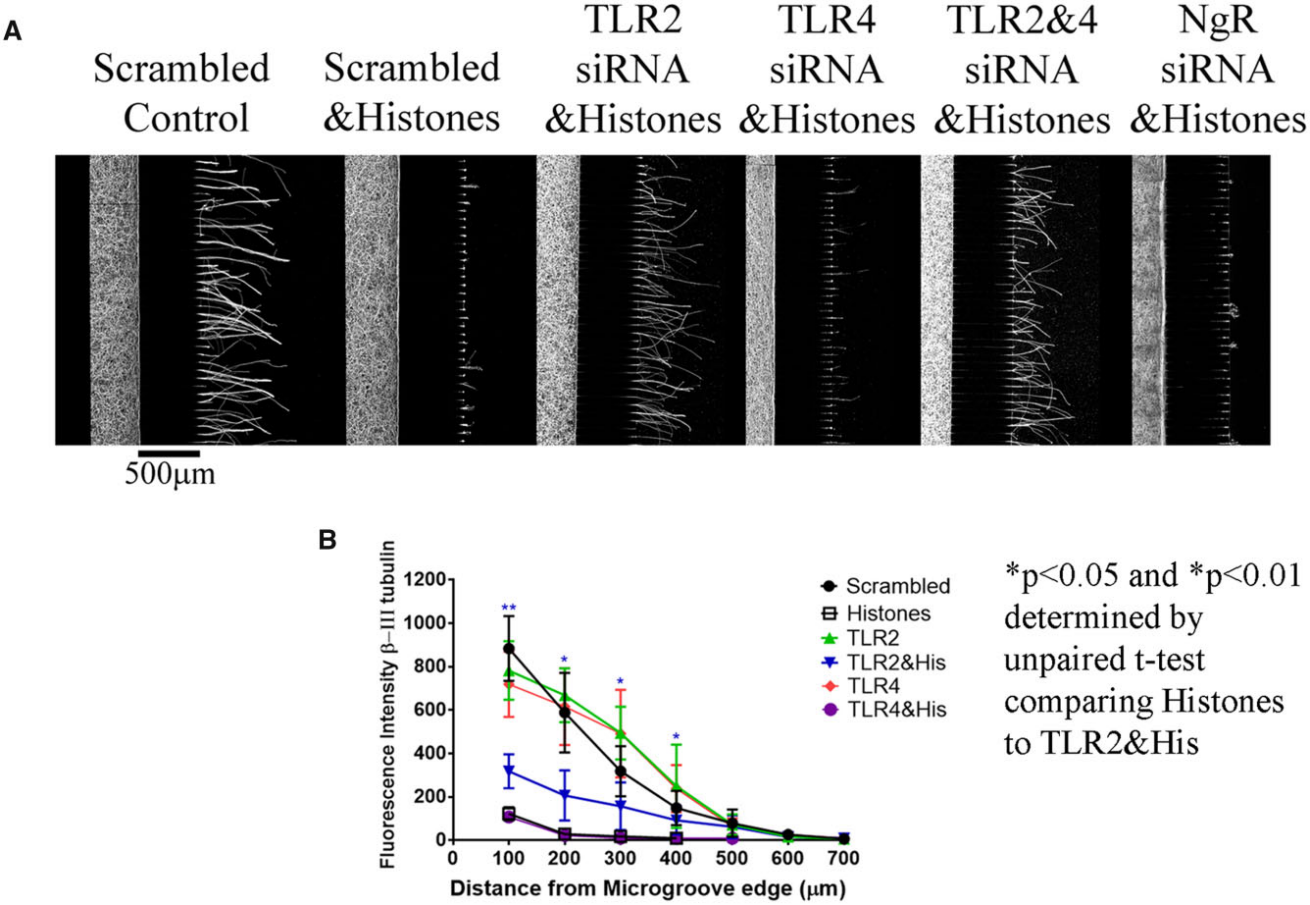
**Toll-like receptor (TLR) 2 knockdown by siRNA provides protection from the inhibitory effect of histones.** (**A**) Neurite outgrowth in chambers using siRNA applied to cell bodies compartment (Left of the microgrooves). Imaged at 20×, micrometer of 500 μm bottom of Scrambled control. Controls are treated with scrambled siRNA, 48 h after siRNA application 20 μg/ml histones were applied to neurite chamber (Right of the microgroove) and incubated for 24 h. Neurites are immunostained with β-III-tubulin and we quantified the neurite length by ImageJ analysis, each condition is *N* = 3. (**B**) Graphs of the average quantified length are shown. Statisitcs are unpaired *t*-tests, where we compared scrambled with Histones to TLR2 siRNA with Histones, and we see a significant difference, *P < 0.05 and **P < 0.01

Interestingly, the neurites treated with the histone-conjugated beads displayed prominent varicosities (Fig. 9B and D), while neurites treated with unconjugated beads were unaffected (Fig. 9A and C). This not only demonstrates that the beads had no toxic effects, but also provides further evidence that histones negatively affect axonal function and integrity, as these varicosities are indicative of disruptions in axonal transport and impending degeneration. These results demonstrate that CNS injury can induce YB-1 phosphorylation in neurons and given our data showing that histone levels are elevated following optic nerve crush (Fig. 8A), it is highly probable that this occurs through histone-mediated retrograde signalling. The induction of neurite degeneration by the histone-coupled beads suggests that the histones are binding to a receptor. The NgR has been shown to bind the 3 major myelin-associated inhibitors, leading to inhibition of neurite outgrowth and growth cone collapse.^28–31^ Using microfluidic chambers, we treated the somal compartments with NgR siRNA, waited 48 h and then added histones to the neurite compartment and waited another 48 h. We found that siRNA knockdown of NgR provided no protection from the effects of histones, as histone-mediated inhibition was comparable to that seen in a scrambled siRNA control (SC) with histone treatment (Fig. 10A). We then tested for other potential receptors. Rodent cortical neurons have been shown to express TLR 2 and 4.^11^ We treated cortical neurons with siRNA for TLR2 and TLR4, either individually or in combination. When the neurite compartment was treated with histones following the knockdown of TLR2, but not TLR4, we observed a partial block of the inhibitory effects of histones (see Fig. 10A and B). To confirm that our siRNAs were working, we performed RT-PCR and found that after 48 h, siRNA for TLR2 reduced TLR2 mRNA levels by nearly 40% compared to SC siRNA-treated cortical neurons. This reduction in TLR2 mRNA persisted for up to 96 h and in some cases increased to nearly 65% reduction, which covers the time frame of our experiment. For TLR4, the siRNA was not as potent resulting in only 20% reduction in mRNA levels compared to SC siRNA-treated cortical neurons after 96 h, with no significant decrease in TLR4 mRNA detected at 48 h. This difference in siRNA performance could account for the lack of effects of siRNA against TLR4 in our neurite outgrowth assays with histones.

## Discussion

Histones lie at the very core of chromatin structure and function, but here we show that they have a very different role outside the nucleus. Histones are released into the extracellular environment following CNS injury, and they act as potent inhibitors of axonal regeneration both *in vitro* and *in vivo*. Extracellular histones produce a degree of inhibition that is comparable to that elicited by myelin-associated inhibitors and CSPGs,^30^^,^^32^^,^^33^ and we therefore propose that histones should be considered a third major class of inhibitory molecules within the CNS. Eliminating inhibitory factors from the CNS environment has been widely used as a strategy to enhance axonal regeneration, but the impact has often been minimal. Triple mutant mice lacking expression of myelin-associated glycoprotein (MAG), Nogo, and oligodendrocyte myelin glycoprotein did not exhibit spontaneous regeneration of corticospinal and raphespinal axons after SCI.^5^ Similarly, elimination of CSPGs through treatment with chondroitinase ABC (chABC) resulted in only modest regeneration of dorsal column and corticospinal axons (maximum of 4 mm) following dorsal column lesions.^34^ The presence and persistence of extracellular histones at the site of injury could therefore account for the limited axonal regeneration that was observed in these studies.

Having established that extracellular histones inhibit axonal regeneration, we studied their mechanism of action, and our observation that APC can block the effects of histones provides some initial insights. Since APC cleaves histones, we propose that histone proteins are directly responsible for mediating inhibition. This conclusion is further supported by our observation that APC cannot overcome inhibition by MAG in a neurite outgrowth assay (Supplementary Fig. 13), which demonstrates that APC does not enhance growth through non-specific degradation of myelin-mediated inhibitors or effects on the neurons themselves. However, we cannot discern the other well-established properties of APC which inhibits the coagulation cascade and exerts anti-inflammatory and apoptotic effects which may also contribute to it being pro-regenerative.^10^

Our results show that histones induce phosphorylation of YB-1 through retrograde signalling which suggests that this effect is receptor-mediated. Receptor ablation experiments show that TLR2, but not NgR or TLR4, mediate the observed effects. Histones have been shown to activate intracellular signalling by binding to cell-surface receptors such as TLR2 and TLR4 in other systems.^12^ It has also been reported that YB-1 can promote microtubule assembly,^35^ and so it is possible that histones may activate RhoA through phosphorylation of YB-1.

The addition of histones to the list of inhibitory molecules acting on the CNS reinforces the prevailing view that combinatorial therapies will be necessary to promote axonal regeneration and functional recovery following SCI. Elevation of intracellular cyclic AMP can overcome inhibition by myelin-associated inhibitors and CSPGs, and we have found that it can reverse inhibition by histones as well (see Supplementary Fig. 1C),^26^^,^^36^ which suggests that rolipram or other phosphodiesterase inhibitors could be an effective way to target all three classes of inhibitors. Histones could also be neutralized using APC, which would digest the histones within the lesion site and create a more permissive environment for regeneration. It should be noted that this treatment would not affect histones in intact cells, which means that APC could be selective, with few adverse effects *in vivo*. This approach could be particularly effective when used in combination with chABC and other interventions that modify the extracellular environment, such as cell transplantation. The findings presented in this study highlight the complexity underlying the challenge of promoting axonal regeneration in the CNS, but they also reveal new opportunities for developing mechanism-based treatments for SCI relevant to humans.

## Supplementary material

Supplementary material is available at *Brain Communications* online.

## Supplementary Material

fcab271_Supplementary_Data

## Abbreviations

APC =: activated protein C
APN =: aprotinin
ChABC =: chondroitinase ABC
CHO =: Chinese hamster ovary
CSPGs =: chondroitin sulphate proteoglycans
CTB =: cholera toxin B-subunit
DCL =: dorsal column lesion
DEGs =: differentially expressed genes
DRG =: dorsal root ganglia
GO =: geneontology
HIS =: histones
LAM =: laminectomy
MAG =: myelin-associated glycoprotein
NgR =: Nogo receptor
ONC =: optic nerve crush
PFA =: paraformaldehyde
SCI =: spinal cord injury
SCPs =: subcellular pathways
T =: thoracic
TLR =: toll-like receptors
YB-1 =: Y-box-binding protein 1

## References

[1] Filbin MT. Myelin-associated inhibitors of axonal regeneration in the adult mammalian CNS. Nat Rev Neurosci. 2003;4(9):703–713. 12951563 10.1038/nrn1195

[2] Buchli AD, Schwab ME. Inhibition of Nogo: A key strategy to increase regeneration, plasticity and functional recovery of the lesioned central nervous system. Ann Med. 2005;37(8):556–567. 16338758 10.1080/07853890500407520

[3] Galtrey CM, Fawcett JW. The role of chondroitin sulfate proteoglycans in regeneration and plasticity in the central nervous system. Brain Res Rev. 2007;54(1):1–18. 17222456 10.1016/j.brainresrev.2006.09.006

[4] He Z, Jin Y. Intrinsic control of axon regeneration. Neuron. 2016;90(3):437–451. 27151637 10.1016/j.neuron.2016.04.022

[5] Cafferty WB, Duffy P, Huebner E, Strittmatter SM. MAG and OMgp synergize with Nogo-A to restrict axonal growth and neurological recovery after spinal cord trauma. J Neurosci. 2010;30(20):6825–6837. 20484625 10.1523/JNEUROSCI.6239-09.2010 PMC2883258

[6] Xu J, Zhang X, Pelayo R, et al Extracellular histones are major mediators of death in sepsis. Nat Med. 2009;15(11):1318–1321. 19855397 10.1038/nm.2053 PMC2783754

[7] Bolton SJ, Russelakis-Carneiro M, Betmouni S, Perry VH. Non-nuclear histone H1 is upregulated in neurons and astrocytes in prion and Alzheimer’s diseases but not in acute neurodegeneration. Neuropathol Appl Neurobiol. 1999;25(5):425–432. 10564533 10.1046/j.1365-2990.1999.00171.x

[8] Gilthorpe JD, Oozeer F, Nash J, et al Extracellular histone H1 is neurotoxic and drives a pro-inflammatory response in microglia. F1000 Res. 2013;2(148):148. 10.12688/f1000research.2-148.v1 PMC3782347 24358859

[9] De Meyer SF, Suidan GL, Fuchs TA, Monestier M, Wagner DD. Extracellular chromatin is an important mediator of ischemic stroke in mice. Arterioscler Thromb Vasc Biol. 2012;32(8):1884–1891. 22628431 10.1161/ATVBAHA.112.250993 PMC3494463

[10] Griffin JH, Fernández JA, Lyden PD, Zlokovic BV. Activated protein C promotes neuroprotection: Mechanisms and translations to the clinic. Thromb Res. 2016;141(Suppl 2):S62–S64. 27207428 10.1016/S0049-3848(16)30368-1 PMC4904825

[11] Tang SC, Arumugam TV, Xu X, et al Pivotal role for neuronal Toll-like receptors in ischemic brain injury and functional deficits. Proc Natl Acad Sci U S A. 2007;104(34):13798–13803. 17693552 10.1073/pnas.0702553104 PMC1959462

[12] Marsman G, Zeerleder S, Luken BM. Extracellular histones, cell-free DNA, or nucleosomes: Differences in immunostimulation. Cell Death Dis. 2016;7(12):e2518. 27929534 10.1038/cddis.2016.410 PMC5261016

[13] Mukhopadhyay G, Doherty P, Walsh FS, Crocker PR, Filbin MT. A novel role for myelin-associated glycoprotein as an inhibitor of axonal regeneration. Neuron. 1994;13(3):757–767. 7522484 10.1016/0896-6273(94)90042-6

[14] Ertürk A, Mauch CP, Hellal F, et al Three-dimensional imaging of the unsectioned adult spinal cord to assess axon regeneration and glial responses after injury. Nat Med. 2011;18(1):166–171. 22198277 10.1038/nm.2600

[15] Mariottini C, Munari L, Gunzel E, et al Wilm’s tumor 1 promotes memory flexibility. Nat Commun. 2019;10(1):3756. 31434897 10.1038/s41467-019-11781-x PMC6704057

[16] Stillitano F, Hansen J, Kong CW, et al Modeling susceptibility to drug-induced long QT with a panel of subject-specific induced pluripotent stem cells. Elife. 2017;6:e19406. 28134617 10.7554/eLife.19406 PMC5279943

[17] Dobin A, Davis CA, Schlesinger F, et al STAR: Ultrafast universal RNA-seq aligner. Bioinformatics. 2013;29(1):15–21. 23104886 10.1093/bioinformatics/bts635 PMC3530905

[18] Trapnell C, Williams BA, Pertea G, et al Transcript assembly and quantification by RNA-seq reveals unannotated transcripts and isoform switching during cell differentiation. Nat Biotechnol. 2010;28(5):511–515. 20436464 10.1038/nbt.1621 PMC3146043

[19] Chen EY, Tan CM, Kou Y, et al Enrichr: Interactive and collaborative HTML5 gene list enrichment analysis tool. BMC Bioinformatics. 2013;14(14):128. 23586463 10.1186/1471-2105-14-128 PMC3637064

[20] Kuleshov MV, Jones Mr Rouillard Ad Fernandez Nf Duan Q, Wang Z, et al Enrichr: A comprehensive gene set enrichment analysis web server 2016 update. Nucleic Acids Res. 2016;44:gkw377. 10.1093/nar/gkw377 PMC4987924 27141961

[21] Karakikes I, Senyei GD, Hansen J, et al Small molecule-mediated directed differentiation of human embryonic stem cells toward ventricular cardiomyocytes. Stem Cells Transl Med. 2014;3(1):18–31. 24324277 10.5966/sctm.2013-0110 PMC3902291

[22] Lehmann M, Fournier A, Selles-Navarro I, et al Inactivation of Rho signaling pathway promotes CNS axon regeneration. J Neurosci. 1999;19(17):7537–7547. 10460260 10.1523/JNEUROSCI.19-17-07537.1999 PMC6782492

[23] He H, Deng K, Siddiq MM, et al Cyclic AMP and polyamines overcome inhibition by myelin-associated glycoprotein through eIF5A-mediated increases in p35 expression and activation of Cdk5. J Neurosci. 2016;36(10):3079–3091. 26961960 10.1523/JNEUROSCI.4012-15.2016 PMC4783503

[24] Freund I, Deutsch M, Sprecher A. Optical second-harmonic microscopy, crossed-beam summation, and small-angle scattering in rat-tail tendon. Biophys J. 1986;50(4):693–712. 3779007 10.1016/S0006-3495(86)83510-X PMC1329848

[25] Vijayaraghavan S, Huq R, Hausman MR. Methods of peripheral nerve tissue preparation for second harmonic generation imaging of collagen fibers. Methods. 2014;66(2):246–255. 23962836 10.1016/j.ymeth.2013.08.012

[26] Cai D, Shen Y, De Bellard M, Tang S, Filbin MT. Prior exposure to neurotrophins blocks inhibition of axonal regeneration by MAG and myelin via a cAMPdependent mechanism. Neuron. 1999;22(1):89–101. 10027292 10.1016/s0896-6273(00)80681-9

[27] Bromberg KD, Ma'ayan A, Neves SR, Iyengar R. Design logic of a cannabinoid receptor signaling network that triggers neurite outgrowth. Science. 2008;320(5878):903–909. 18487186 10.1126/science.1152662 PMC2776723

[28] Fournier AE, GrandPre T, Strittmatter SM. Identification of a receptor mediating Nogo-66 inhibition of axonal regeneration. Nature. 2001;409(6818):341–346. 11201742 10.1038/35053072

[29] Liu BP, Fournier A, GrandPre T, Strittmatter SM. Myelin-associated glycoprotein as a functional ligand for the Nogo-66 receptor. Science. 2002;297(5584):1190–1193. 12089450 10.1126/science.1073031

[30] Domeniconi M, Cao Z, Spencer T, et al Myelin-associated glycoprotein interacts with the Nogo66 receptor to inhibit neurite outgrowth. Neuron. 2002;35(2):283–290. 12160746 10.1016/s0896-6273(02)00770-5

[31] Oertle T, van der Haar ME, Bandtlow CE, et al Nogo-A inhibits neurite outgrowth and cell spreading with three discrete regions. J Neurosci. 2003;23(13):5393–5406. 12843238 10.1523/JNEUROSCI.23-13-05393.2003 PMC6741224

[32] Shen Y, Tenney AP, Busch SA, et al PTPσ is a receptor for chondroitin sulfate proteoglycan, an inhibitor of neural regeneration. Science. 2009;326(5952):592–596. 19833921 10.1126/science.1178310 PMC2811318

[33] Siddiq MM, Hannila SS, Carmel JB, et al Metallothionein-I/II promotes axonal regeneration in the central nervous system. J Biol Chem. 2015;290(26):16343–16356. 25947372 10.1074/jbc.M114.630574 PMC4481232

[34] Bradbury EJ, Moon LD, Popat RJ, et al Chondroitinase ABC promotes functional recovery after spinal cord injury. Nature. 2002;416(6881):636–640. 11948352 10.1038/416636a

[35] Chernov KG, Mechulam A, Popova NV, et al YB-1 promotes microtubule assembly in vitro through interaction with tubulin and microtubules. BMC Biochem. 2008;9:23. 18793384 10.1186/1471-2091-9-23 PMC2557009

[36] Lu P, Yang H, Jones LL, Filbin MT, Tuszynski MH. Combinatorial therapy with neurotrophins and cAMP promotes axonal regeneration beyond sites of spinal cord injury. J. Neurosci. 2004;24(28):6402–6409. 15254096 10.1523/JNEUROSCI.1492-04.2004 PMC6729552

