# Supplementary material for: Extracellular histones, a new class of inhibitory molecules of CNS axonal regeneration
Source: Brain Commun. 2021 Nov 13;3(4):fcab271. doi: 10.1093/braincomms/fcab271 (PMC8728726; doi:10.1093/braincomms/fcab271)
Supplement: fcab271_Supplementary_Data [file fcab271_Supplementary_Data.zip › LegendsSupplementaryFinal.docx]

Extracellular histones, a new class of inhibitory molecules of CNS axonal regeneration

Short title: Histones inhibit axonal regeneration through TLR2

Mustafa M. Siddiq^1,2^, Sari S. Hannila^1,3^, Yana Zorina^2,4^, Elena Nikulina^1,5^, Vera Rabinovich^2^,

Jianwei Hou^1^ , Rumana Huq^2^, Erica L. Richman^1^, Rosa E. Tolentino^2^, Jens Hansen^2^, Adam Velenosi^6^, Brian K. Kwon^7^, Stella E. Tsirka^8^, Ian Maze^9^, Robert Sebra^10,11,12^, Kristin G. Beaumont^10,11^, Carlos A. Toro^13,14^, Christopher P. Cardozo^13,14,15^, Ravi Iyengar^2^* and Marie T. Filbin^1^*#

**Supplementary Figure Legends**

**Supp. Figure S1 – Extracellular histones inhibit neurite outgrowth on a permissive substrate CHO monolayers.**

1. Representative images showing the whole field imaged and quantitated. These are cortical neurons on CHO monolayers stained with β-III tubulin and a dose-dependent inhibition of neurite outgrowth by mixed population of histones from calf thymus (5-20$\mu g/ml)$ was observed.
2. Representative images of cortical neurons on CHO monolayers stained with β-III tubulin and a dose-dependent inhibition of neurite outgrowth by mixed population of histones from calf thymus (5-20$\mu g/ml)$ and histones applied with 1mM dbc-AMP, where dbc-AMP can overcome the inhibitory effect to neurite outgrowth.
3. Quantification for the images in Figure S1B, each bar is the average of three independent experiments. For statistics we used one way ANOVA with Bonferroni multiple comparison test and compared all treatments to control, no treatment, **p<0.01.

**Supp. Figure S2. Extracellular histones are inhibitory to neurite outgrowth and braching in primary dorsal root ganglion.** Dorsal root ganglion (DRG) neurons grow shorter neurites and have fewer branches in the presence of extracellular histones. P5 rat DRG neurons are plated on a permissive layer of CHO monolayers with or without extracellular histones. Histone-treatment results in shorter neurites and we also quantified the average number of branches which was significantly reduced in the presence of histones. All statistics are performed by one way ANOVA with Bonferroni multiple comparison test, **p<0.01.

**Supp. Figure S3 - Extracellular histones inhibit neurite outgrowth on a permissive GFAP+ Astrocytic monolayer and do not affect CSPG secretion.** A. Primary rat astrocytes were grown on PLL-coated glass 8 wells microscope slides to confluency. We applied histones in the concentrations indicated and added cortical neurons at the same time. We incubated for 24hrs, stained for β-III-tubulin and observed a dose-dependent inhibition of neurite outgrowth. B. Astrocyte cultures were treated with either mixed histone population or with TGF-β, which is known to increase secretion of CSPGs. Compared to TGF-β, histones did not up-regulate secretion of either neurocan or brevican.

**Supp. Figure S4 – Recombinant H3 and H4 histone isoforms are inhibitory to neurite outgrowth and contribute to dystrophic bulb formation.** Using microfluidic chambers with cortical neurons treated with either recombinant H3 or H4 isoforms, we observed that H3 had a more robust effect on inhibiting neurite outgrowth and promoted dystrophic bulb formation. H4 also resulted in slightly shorter neurites and promoted dystrophic bulb formation.

**Supp. Figure S5 – Extracellular histones are inhibitory to neurite outgrowth on PLL-coated microfluidic chambers.** Using PLL-coated microfluidic chambers, cortical neurons cell bodies (CB) grow long neurites across the 450µM microgroove as detected by β-III tubulin. Treating the neurite growing compartment with either recombinant H3 or H4 Histones resulted in significantly shorter neurites, with H3 having a more potent effect. Representative images showing the full chamber.

**Supp. Figure S6 –Mixed population of histone are inhibitory to neurite outgrowth in a dose-dependent fashion.** Cortical neurons plated on PLL coated microfluidic chambers grow robustly across the 450µm microgroove, determined by β-III tubulin staining. Treating the neurite growing side (right side from the microgroove) only with increasing concentrations of a mixed population of histones isolated from calf thymus results in significantly shorter neurites with the following conditions: Control, Aprotinin-treated, Histones - 5µg/ml, Histones 10(µg/ml) and Histones 20(µg/ml).

**Supp. Figure S7 – Activated Protein C (APC) blocks the inhibitory effect of Histones.** APC alone has no effect on neurite outgrowth, combining APC with histones and applying to the neurite side reverses the inhibitory effect of histones, restoring long neurite outgrowth in microfluidic chambers.

**Supp. Figure S8 - Volumetric analysis in nerves treated with APC after ONC.** A. APC-treated nerve, for representation we show how we measure the Normalized Segmented Area Ratio. The cross section slice is set at the middle of the crush site (vertical red bar demarcating). B. The graph representing the Normalized Segmented Area Ratio for the image in A.

**Supp. Figure S9 – APC treatment in the optic nerve crush using Volocity 3D Projection.** Representative ONC treated with APC at the injury site, promoting axonal regeneration. Using Volocity to get a 3D projection of the same nerve to reveal axons regenerating within the nerve.

**Supp. Figure S10 - Priming cortical neurons with histones results in significantly shorter neurites.** Cortical neurons are treated overnight with either PBS (Con) or 5, 10 or 20µg/ml of mixed prep of histones. The neurons are aspirated and washed with plain NB, trypsinized and plated onto a confluent monolayer of permissive CHO cells in supplemented NB. Note there are no histones subsequently added once on the CHO cells, histones are only present during the overnight priming and then washed out. Once fixed and immunostained with β-III tubulin and quantified, we observed significantly shorter neurites compared to the control (**p<0.01).

**Supp. Figure S11 - The transcription factor YB-1 is elevated in response to histone treatment on primary cortical neurons.** Using a Panomics DNA-protien binding array we observed elevation in the transcription factor YB-1 in primary cortical neurons. We treated our samples with PBS or histones for 30 or 120 minutes before preparing nuclear extracts used in a commercial assay for transcription factor activation. With histone treatment we saw elevation in YB-1 levels as shown in the blowups and circled.

**Supp. Figure S12 – Extracellular histones elevate pYB-1 in cortical neurons.** Normalized values from Figure 4B western blots, where we treated cortical neurons for 2hrs with either a mixed population of histones (Histone), recombinant H3 or H4 at 2.5, 5 or 10µg/ml. The graph shows normalized values of pYB-1 to total YB-1.

**Supp. Figure S13- APC does not overcome MAG-mediated inhibition.** Cortical neurons are plated on either control CHO cells or Myelin-Associated Glycoprotein (MAG)-expressing CHO cells. On MAG, cortical neurons are inhibited from putting out neurites. APC application to the neurons when plated on MAG does not overcome this inhibition unlike dbcAMP which is known to overcome myelin-mediated inhibitors.

**Supp. Figures S14-33 are the full non-cropped gels that are included in the manuscript:**

**Supp. Figure S14 - Elevated levels of extracellular histones are detected in the injured human CNS probing with antibody for Histone H3.** Non-cropped gel presented in Fig. 1A. From human CSF collected from patients approximately 24 hrs post-injury with ASIA score of A (complete impairment, paralysis), and the controls are all normal (ASIA Grade E). On a gradient Western blot, we loaded 30µgs of sample for each human specimen and probed for histone H3. The H3 is at the predicted size of 17kDa and we detect it in patients with Thoracic injury (T6, T10 and T4).

**Supp. Figure S15 - Elevated levels of extracellular histones detected in the injured human CNS probing with antibody for Albumin to normalize samples.** Non-cropped gel presented in Fig. 1A. From human CSF collected from patients approximately 24 hrs post-injury with ASIA score of A (complete impairment, paralysis), and the controls are all normal (ASIA Grade E). On a gradient Western blot, we loaded 30µgs of sample for each human specimen and probed for Albumin (ALB) for normalizing control.

**Supp. Figure S16 - Elevated levels of extracellular histones detected in the injured mouse CNS probing with antibody for H3.** Non-cropped gel presented in Fig. 1C. Elevated levels of Histone H3 (HIS) in the CSF fluid collected from mice with DCL are detected by Western blot, compared to laminectomy (LAM) alone.

**Supp. Figure S17 - Elevated levels of extracellular histones detected in the injured mouse CNS probing with antibody for Albumin to normalize samples.** Non-cropped gel presented in Fig. 1C. ALB levels in the CSF fluid collected from mice with DCL and LAM alone detected by Western blot.

**Supp. Figure S18 - Elevated levels of extracellular histones detected in the injured rat CNS probing with antibody for Histone H3.** Non-cropped gel presented in Fig. 1E. Adult rats first had their optic nerves exposed and we applied gelfoam over the uninjured nerve for 48hrs (Pre-ONC1&2), subsequently removing the gelfoam into PBS with protease inhibitor cocktail, we crushed the optic nerve and applied a fresh piece of gelfoam over the injury site. We removed the gelfoam 48hrs later (Post-ONC 1&2). We found within the same animal a significant increase in HIS levels after crushing the optic nerve (Post-ONC) than prior to injury (Pre-ONC).

**Supp. Figure S19 - Elevated levels of extracellular histones detected in the injured rat CNS probing with antibody for Albumin to normalize samples.** Non-cropped gel presented in Fig. 1E. Adult rats first had their optic nerves exposed and we applied gelfoam over the uninjured nerve for 48hrs (Pre-ONC1&2), subsequently removing the gelfoam into PBS with protease inhibitor cocktail, we crushed the optic nerve and applied a fresh piece of gelfoam over the injury site. We removed the gelfoam 48hrs later (Post-ONC 1&2). We probed for Albumin detected by western blot.

**Supp. Figure S20 – Extracellular Histones activate Rho in cortical neurons.** Non-cropped gel presented in Fig. 3B from a Rho pull-down assay. Histone applied to cortical neurons induces activation of Rho GTPase, with N = 5, one sample per condition that was used in 5 different experiments. Extracellular histones applied to cortical neurons do activate Rho, as detected in the Rho pull-down assay.

**Supp. Figure S21 – Extracellular Histones activate Rho in cortical neurons, normalized for total Rho.** Non-cropped gel presented in Fig. 3B for total Rho. Histone applied to cortical neurons induces activation of Rho GTPase, with N = 5, one sample per condition that was used in 5 different experiments. Extracellular histones applied to cortical neurons and probed for Rho by western blot.

**Supp. Figure S22 - Extracellular Histones decrease p35 levels.** Non-cropped gel presented in Fig. 3C probing for p35 by Western blot. Histone applied to cortical neurons results in dose dependent decrease (2.5-20µg/ml) in p35 levels without any detectable p25.

**Supp. Figure S23 - Extracellular Histones decrease p35 levels normalized with Actin.** Non-cropped gel presented in Fig. 3C probing for Actin by Western blot in cortical neurons.

**Supp. Figure S24 -** **Histones inhibit neurite outgrowth by an YB-1 mediated mechanism.** Non-cropped gel presented in Fig. 8A probing for in phosphorylated YB-1 (pYB-1). To confirm if pYB-1 are elevated after injury inducing histone release in the ONC, we crushed the axons of the optic nerve and 48hrs later collected the retinal cell layer, ipsilateral side (Ipsi 1&2), and collected the retinal layer of the non-injured, contralateral side (Con1&2) in the same animal and prepared them for Western blots. We found significantly elevated levels of pYB-1 normalized to total YB-1 on the injured (Ipsi) side compared to the non-injured (Contra) side.

**Supp. Figure S25 -** **Histones inhibit neurite outgrowth by an YB-1 mediated mechanism.** Non-cropped gel presented in Fig. 8A probing for Total YB-1. We crushed the axons of the optic nerve and 48hrs later collected the retinal cell layer, ipsilateral side (Ipsi 1&2), and collected the retinal layer of the non-injured, contralateral side (Con1&2) in the same animal and prepared them for Western blots probing for Total YB-1.

**Supp. Figure S26 -** **Histones inhibit neurite outgrowth by an YB-1 mediated mechanism.** Non-cropped gel presented in Fig. 8A probing for β-III tubulin for normalization of samples. We crushed the axons of the optic nerve and 48hrs later collected the retinal cell layer, ipsilateral side (Ipsi 1&2), and collected the retinal layer of the non-injured, contralateral side (Con1&2) in the same animal and prepared them for Western blots probing for β-III tubulin.

**Supp. Figure S27 -** **Histones inhibit neurite outgrowth by an YB-1 mediated mechanism in rat cortical neurons.** Non-cropped gel presented in Fig. 8B probing for pYB-1. Cortical neurons treated with mixed Histones, H3 or H4 (2.5,5, or 10µg/ml) induce elevation in pYB-1, in a dose dependent fashion.

**Supp. Figure S28 -** **Histones inhibit neurite outgrowth by an YB-1 mediated mechanism in rat cortical neurons.** Non-cropped gel presented in Fig. 8B probing for Total YB-1. Cortical neurons treated with mixed Histones, H3 or H4 (2.5,5, or 10µg/ml) and then probed for YB-1 by western blot.

**Supp. Figure S29 -** **Histones inhibit neurite outgrowth by an YB-1 mediated mechanism in rat cortical neurons normalized to Actin.** Non-cropped gel presented in Fig. 8B probing for Actin. Cortical neurons treated with mixed Histones, H3 or H4 (2.5,5, or 10µg/ml) and then probed for Actin by western blot, for normalization of samples.

**Supp. Figure S30 – Histones mediated activation of pYB-1 attenuated by APC.** Non-cropped gel presented in Fig. 8C probing for pYB-1. In cortical neurons treated with 10μg/ml mixed Histones (H10) we see up-regulation of pYB-1. APC does not significantly alter phosphorylation of YB-1 but it does attenuate Histone-mediated activation of YB-1 compared to Control.

**Supp. Figure S31 – Histones mediated activation of pYB-1 attenuated by APC, normalized to Actin.** Non-cropped gel presented in Fig. 8C probing for Actin to normalize the samples.

**Supp. Figure S32 -** **Histones inhibit neurite outgrowth by a retrograde pYB-1 mediated mechanism in rat cortical neurons.** Non-cropped gel presented in Fig. 8E probing for pYB-1. Using histones or fluorescent microbeads that are covalently coupled to histones (or non-coupled for controls) and too large to cross the microgrooves, or with 10 or 20 µg/ml mixed Histones (His10 or His20) are applied to the neurite compartment (axonal) only and we wait 48hrs before lysing the cell body compartment (somal) with 2XRIPA buffer and run a Western blot. We detect elevation of pYB-1 in the cell bodies normalized to GAPDH.

**Supp. Figure S33 -** **Histones inhibit neurite outgrowth by a retrograde pYB-1 mediated mechanism in rat cortical neurons.** Non-cropped gel presented in Fig. 8E probing for GAPDH. Using histones or fluorescent microbeads that are covalently coupled to histones (or non-coupled for controls) and too large to cross the microgrooves, or with 10 or 20 µg/ml mixed Histones (His10 or His20) are applied to the neurite compartment (axonal) only and we wait 48hrs before lysing the cell body compartment (somal) with 2XRIPA buffer and run a Western blot. We detect elevation of pYB-1 in the cell bodies normalized to GAPDH.
