# Supplementary material for: Extracellular histones, a new class of inhibitory molecules of CNS axonal regeneration
Source: Brain Commun. 2021 Nov 13;3(4):fcab271. doi: 10.1093/braincomms/fcab271 (PMC8728726; doi:10.1093/braincomms/fcab271)

Extracellular histones, a new class of inhibitory molecules of CNS axonal regeneration

Short title: Histones inhibit axonal regeneration through TLR2

Mustafa M. Siddiq[1,2], Sari S. Hannila[1,3], Yana Zorina[2,4], Elena Nikulina[1,5], Vera Rabinovich[2], Jianwei Hou[1] , Rumana Huq[2], Erica L. Richman[1], Rosa E. Tolentino[2], Jens Hansen[2], Adam Velenosi[6], Brian K. Kwon[7], Stella E. Tsirka[8], Ian Maze[9], Robert Sebra[10,11,12], Kristin G. Beaumont[10,11], Carlos A. Toro[13,14], Christopher P. Cardozo[13,14,15], Ravi Iyengar[2]* and Marie T. Filbin[1]*#

Supplementary

Figure S1 – Extracellular histones inhibit neurite outgrowth on a permissive substrate CHO monolayers.

A. Representative images showing the whole field imaged and quantitated. These are cortical neurons on CHO monolayers stained with β-III tubulin and a dose-dependent inhibition of neurite outgrowth by mixed population of histones from calf thymus (5-20μg/ml) was observed.
B. Representative images of cortical neurons on CHO monolayers stained with β-III tubulin and a dose-dependent inhibition of neurite outgrowth by mixed population of histones from calf thymus (5-20μg/ml) and histones applied with 1mM dbc-AMP, where dbc-AMP can overcome the inhibitory effect to neurite outgrowth.
C. Quantification for the images in Figure S1B, each bar is the average of three independent experiments. For statistics we used one way ANOVA with Bonferroni multiple comparison test and compared all treatments to control, no treatment, $**p<0.01$.

Figure S2. Extracellular histones are inhibitory to neurite outgrowth and braching in primary dorsal root ganglion. Dorsal root ganglion (DRG) neurons grow shorter neurites and have fewer branches in the presence of extracellular histones. P5 rat DRG neurons are plated on a permissive layer of CHO monolayers with or without extracellular histones. Histone-treatment results in shorter neurites and we also quantified the average number of branches which was significantly reduced in the presence of histones. All statistics are performed by one way ANOVA with Bonferroni multiple comparison test, $**p<0.01$.

Figure S3 - Extracellular histones inhibit neurite outgrowth on a permissive GFAP+ Astrocytic monolayer and do not affect CSPG secretion. A. Primary rat astrocytes were grown on PLL-coated glass 8 wells microscope slides to confluency. We applied histones in the concentrations indicated and added cortical neurons at the same time. We incubated for 24hrs, stained for β-III-tubulin and observed a dose-dependent inhibition of neurite outgrowth. B. Astrocyte cultures were treated with either mixed histone population or with TGF-β, which is known to increase secretion of CSPGs. Compared to TGF-β, histones did not up-regulate secretion of either neurocan or brevican.

Figure S4 – Recombinant H3 and H4 histone isoforms are inhibitory to neurite outgrowth and contribute to dystrophic bulb formation. Using microfluidic chambers with cortical neurons treated with either recombinant H3 or H4 isoforms, we observed that H3 had a more robust effect on inhibiting neurite outgrowth and promoted dystrophic bulb formation. H4 also resulted in slightly shorter neurites and promoted dystrophic bulb formation.

Figure S5 – Extracellular histones are inhibitory to neurite outgrowth on PLL-coated microfluidic chambers. Using PLL-coated microfluidic chambers, cortical neurons cell bodies (CB) grow long neurites across the 450μM microgroove as detected by β-III tubulin. Treating the neurite growing compartment with either recombinant H3 or H4 Histones resulted in significantly shorter neurites, with H3 having a more potent effect. Representative images showing the full chamber.

Figure S6 –Mixed population of histone are inhibitory to neurite outgrowth in a dose-dependent fashion. Cortical neurons plated on PLL coated microfluidic chambers grow robustly across the 450μm microgroove, determined by β-III tubulin staining. Treating the neurite growing side (right side from the microgroove) only with increasing concentrations of a mixed population of histones isolated from calf thymus results in significantly shorter neurites with the following conditions: Control, Aprotinin-treated, Histones - 5μg/ml, Histones 10(μg/ml) and Histones 20(μg/ml).

Figure S7 – Activated Protein C (APC) blocks the inhibitory effect of Histones. APC alone has no effect on neurite outgrowth, combining APC with histones and applying to the neurite side reverses the inhibitory effect of histones, restoring long neurite outgrowth in microfluidic chambers.

Figure S8 - Volumetric analysis in nerves treated with APC after ONC. A. APC-treated nerve, for representation we show how we measure the Normalized Segmented Area Ratio. The

cross section slice is set at the middle of the crush site (vertical red bar demarcating). B. The graph representing the Normalized Segmented Area Ratio for the image in A.

Figure S9 – APC treatment in the optic nerve crush using Volocity 3D Projection. Representative ONC treated with APC at the injury site, promoting axonal regeneration. Using Volocity to get a 3D projection of the same nerve to reveal axons regenerating within the nerve.

Figured S10 - Priming cortical neurons with histones results in significantly shorter neurites. Cortical neurons are treated overnight with either PBS (Con) or 5, 10 or 20μg/ml of mixed prep of histones. The neurons are aspirated and washed with plain NB, trypsinized and plated onto a confluent monolayer of permissive CHO cells in supplemented NB. Note there are no histones subsequently added once on the CHO cells, histones are only present during the overnight priming and then washed out. Once fixed and immunostained with β-III tubulin and quantified, we observed significantly shorter neurites compared to the control (**$p<0.01$).

Figure S11 - The transcription factor YB-1 is elevated in response to histone treatment on primary cortical neurons. Using a Panomics DNA-protien binding array we observed elevation in the transcription factor YB-1 in primary cortical neurons. We treated our samples with PBS or histones for 30 or 120 minutes before preparing nuclear extracts used in a commercial assay for transcription factor activation. With histone treatment we saw elevation in YB-1 levels as shown in the blowups and circled.

Figure S12 – Extracellular histones elevate pYB-1 in cortical neurons. Normalized values from Figure 4B western blots, where we treated cortical neurons for 2hrs with either a mixed population of histones (Histone), recombinant H3 or H4 at 2.5, 5 or 10μg/ml. The graph shows normalized values of pYB-1 to total YB-1.

Figure S13- APC does not overcome MAG-mediated inhibition. Cortical neurons are plated on either control CHO cells or Myelin-Associated Glycoprotein (MAG)-expressing CHO cells. On MAG, cortical neurons are inhibited from putting out neurites. APC application to the neurons when plated on MAG does not overcome this inhibition unlike dbcAMP which is known to overcome myelin-mediated inhibitors.

Figures S14-32 are the full length gels that are included in the manuscript.

Supplementary Figure 1A

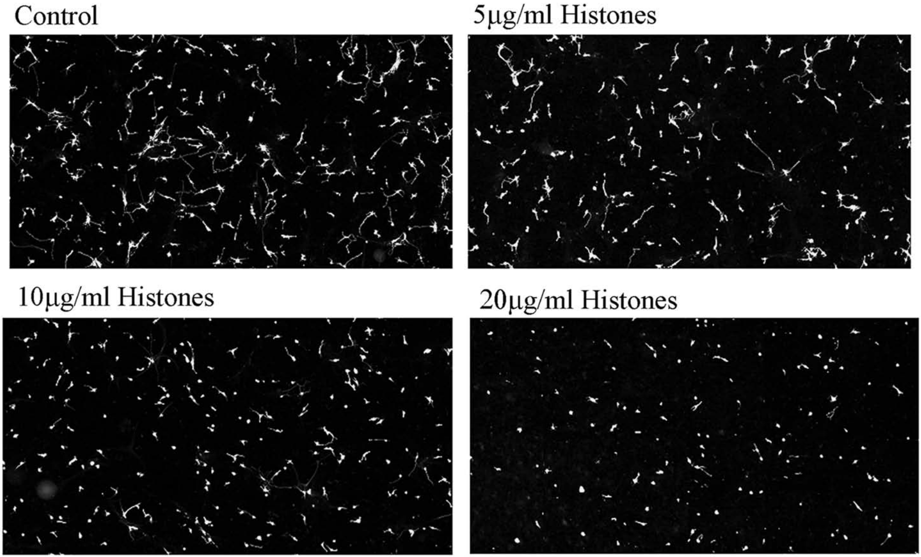

Supplementary Fig 1B

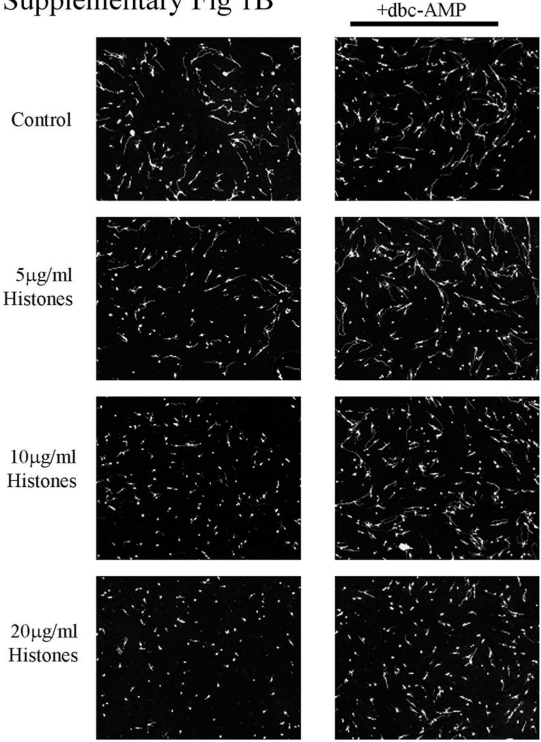

Figure S1C

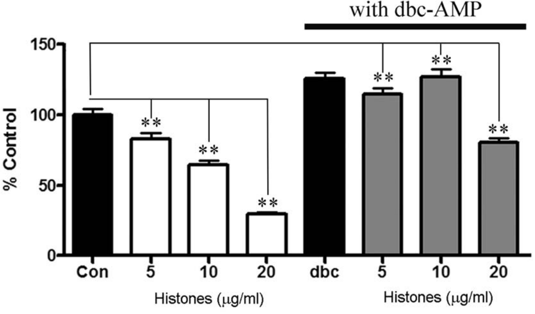

with dbc-AMP
150
100
50
0
% Control
**
**
**
**
**
**
Con
5
10
20
dbc
5
10
20
Histones (μg/ml)
Histones (μg/ml)

Supplementary Figure 2

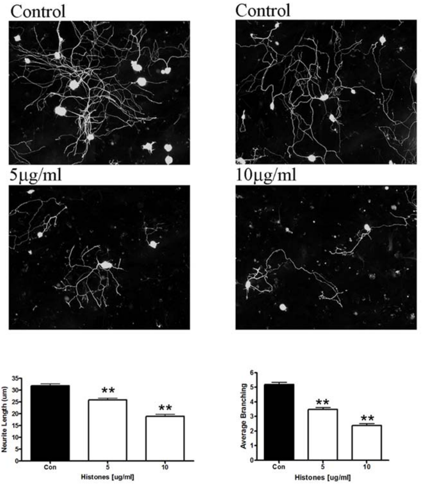

Supplementary Figure 3

S3A.

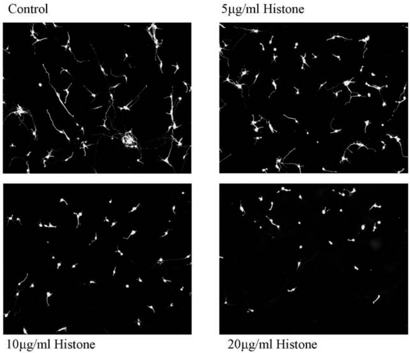

S3B.

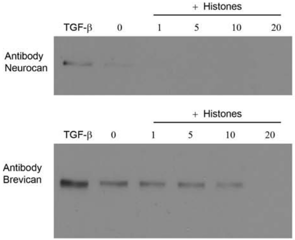

Supplementary Figure 4

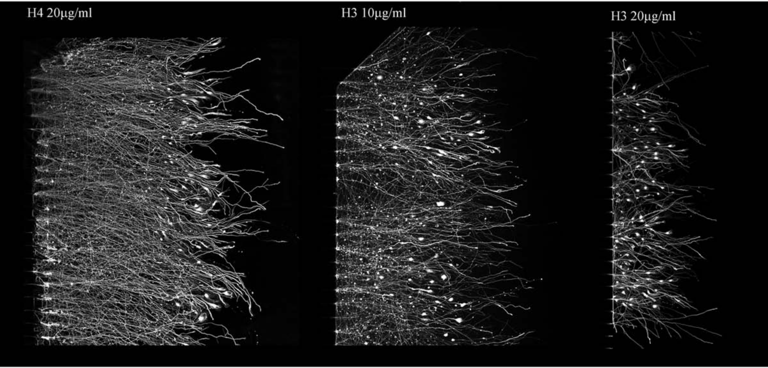

Supplementary Figure 5

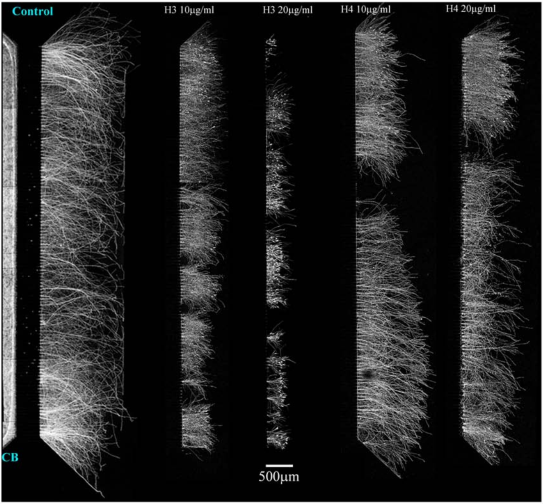

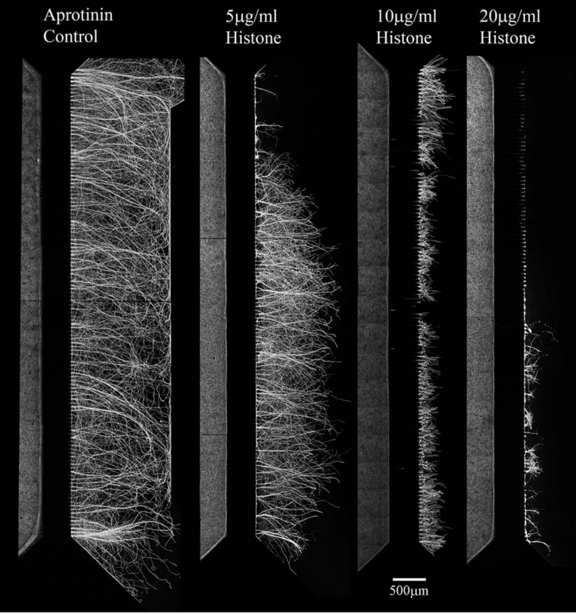

Supplementary
Figure 6

Supplementary Figure 7

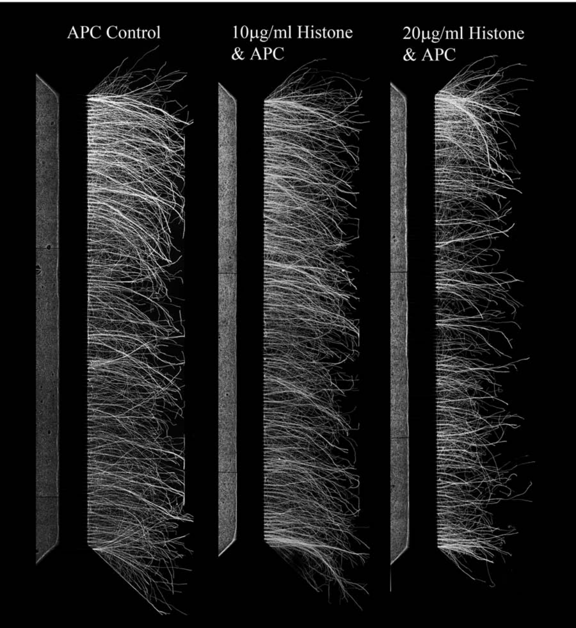

Supplementary Figure 8

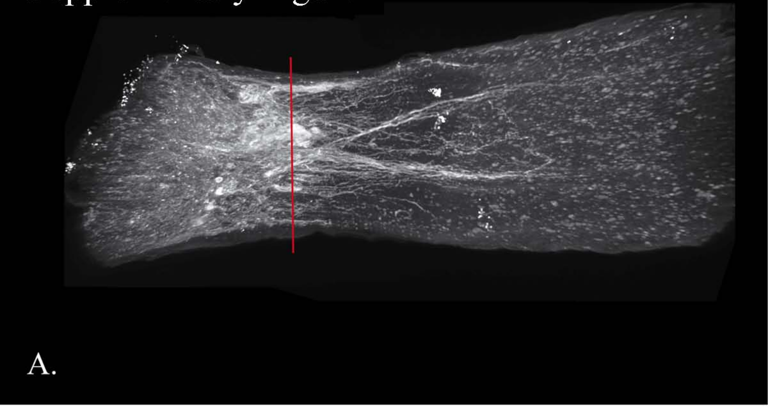

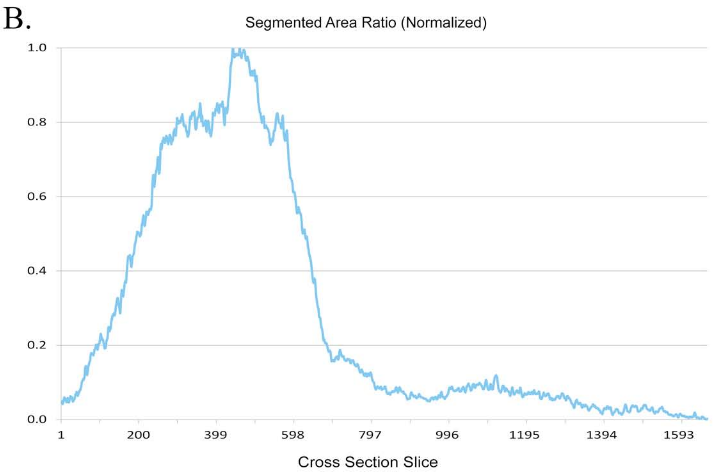

Supplementary Figure 9

APC-treated

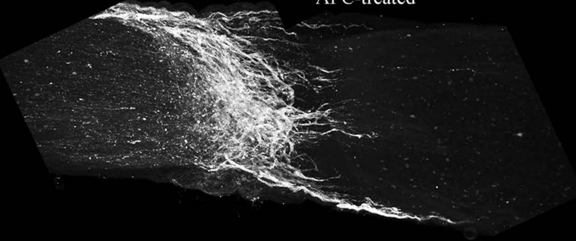

3-Dimensional Projection using Volocity

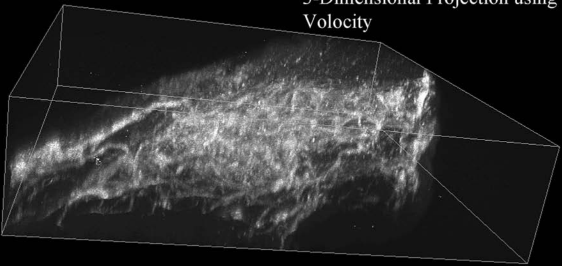

Supplementary Figure 10

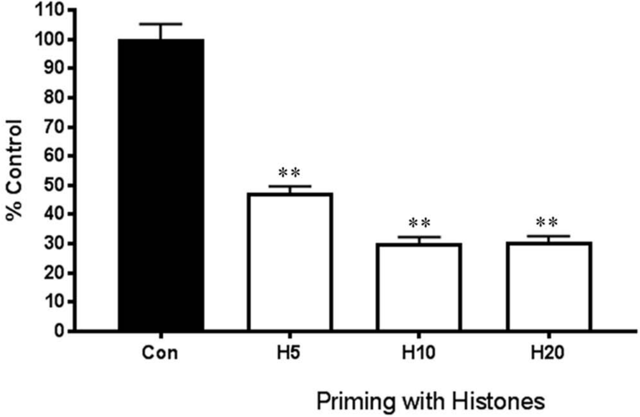

Supplementary Figure 11

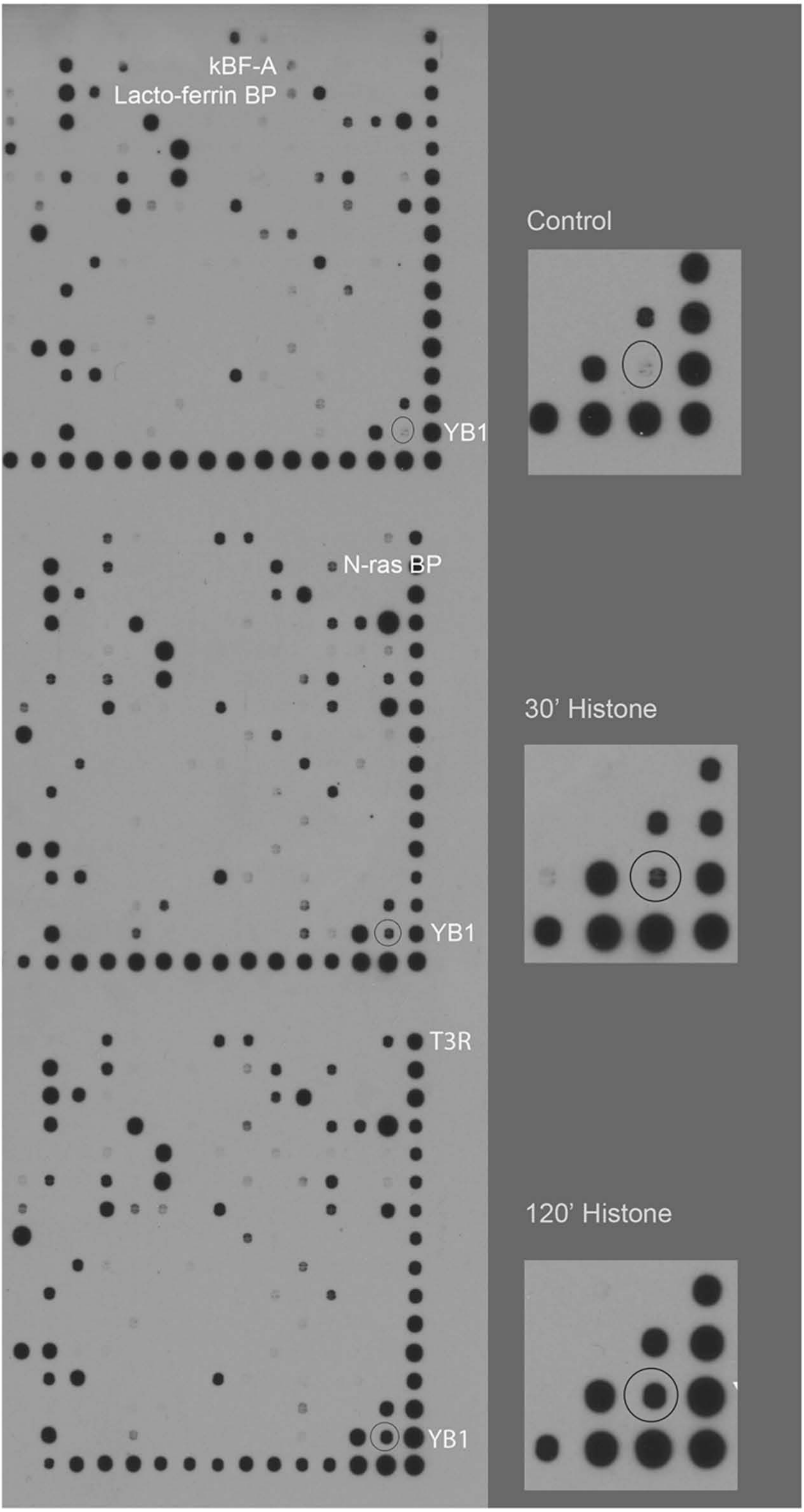

Supplementary Figure 12

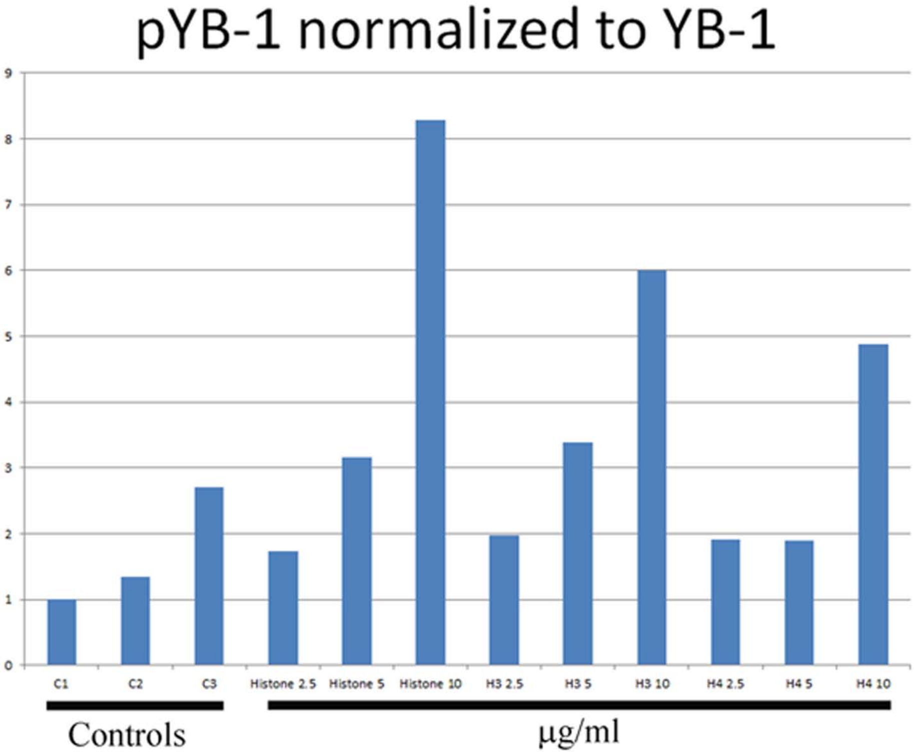

Supplementary Figure 13

A. Control

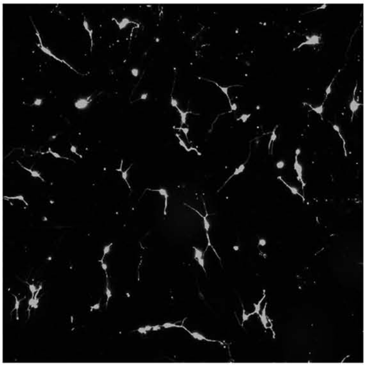

B. MAG

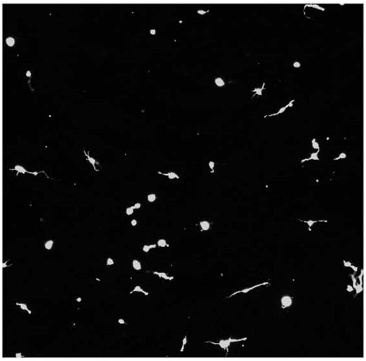

C. MAG with APC

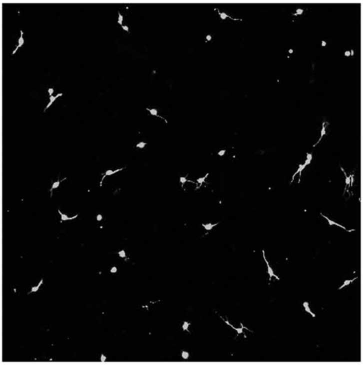

D. MAG with dbcAMP

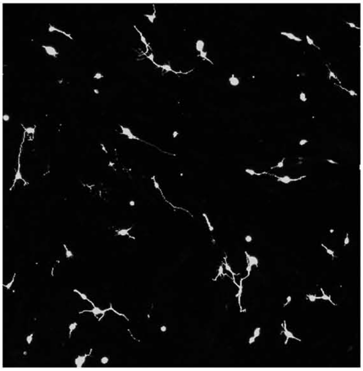

E.

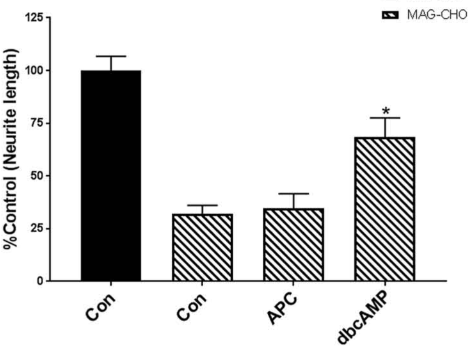

FigS14 - From Fig. 1A using antibody for Histone H3

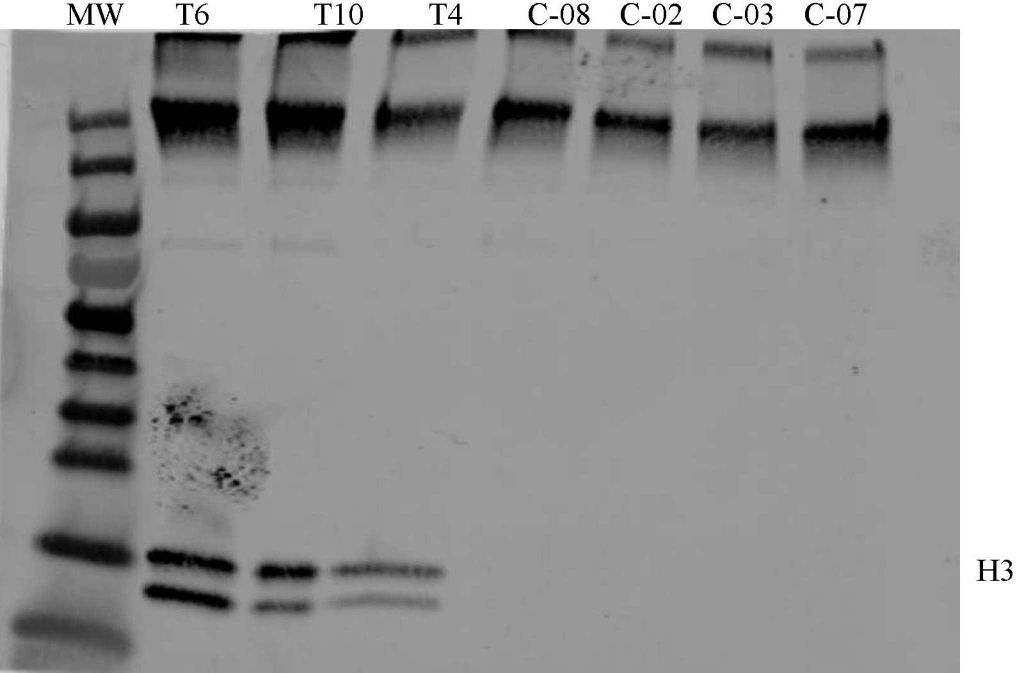

Fig. S15 - From Fig. 1A probing for Albumin antibody

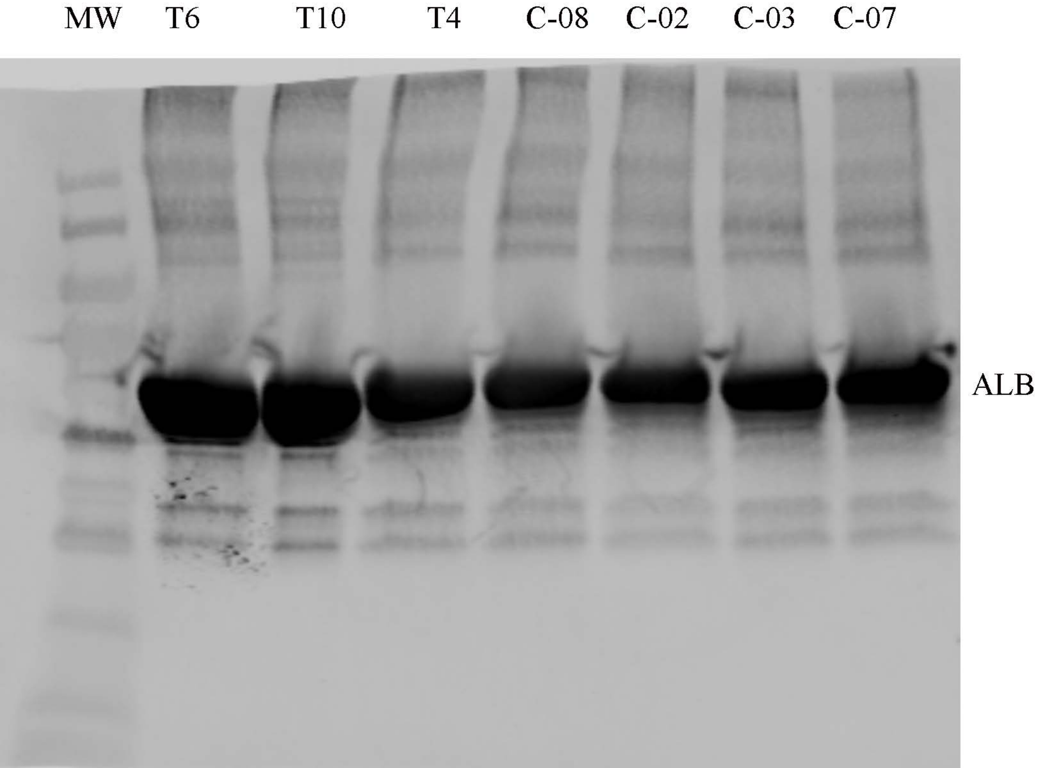

Fig. S16 - Fig. 1C probing for Histone H3

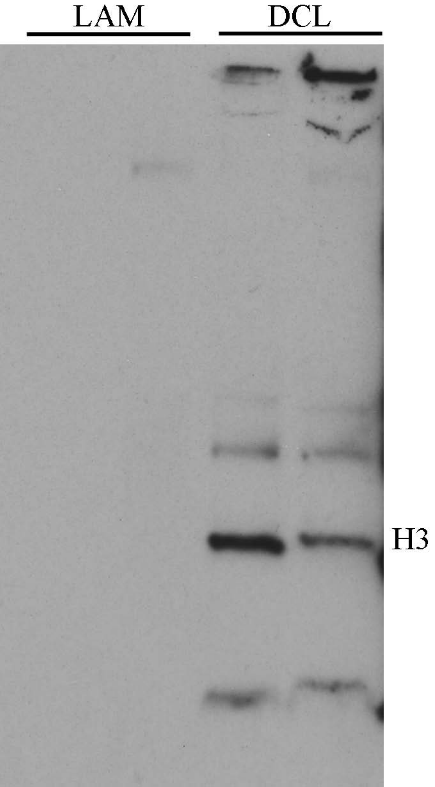

Fig. S17 - Fig. 1C probing with Albumin antibody

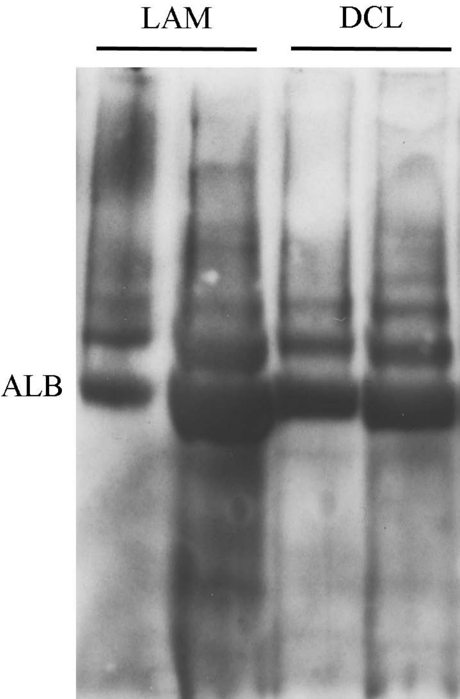

Fig. S18 - from Fig. 1E probing with Histone H3

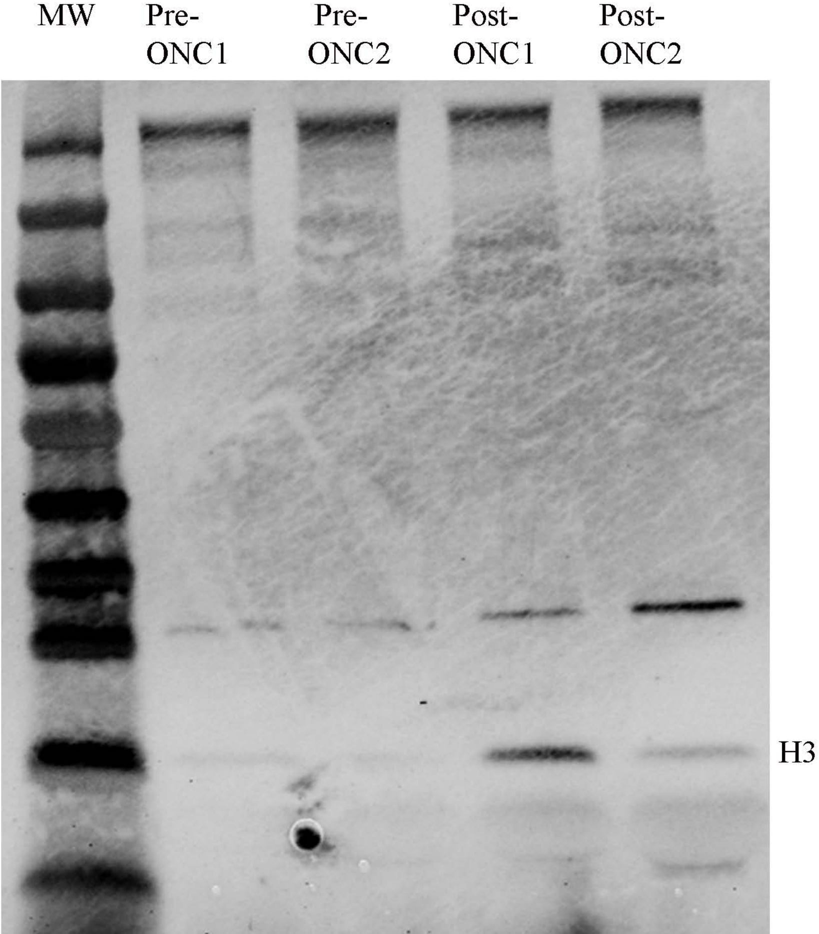

Fig. S19 from Fig. 1E probing with Albumin Antibody

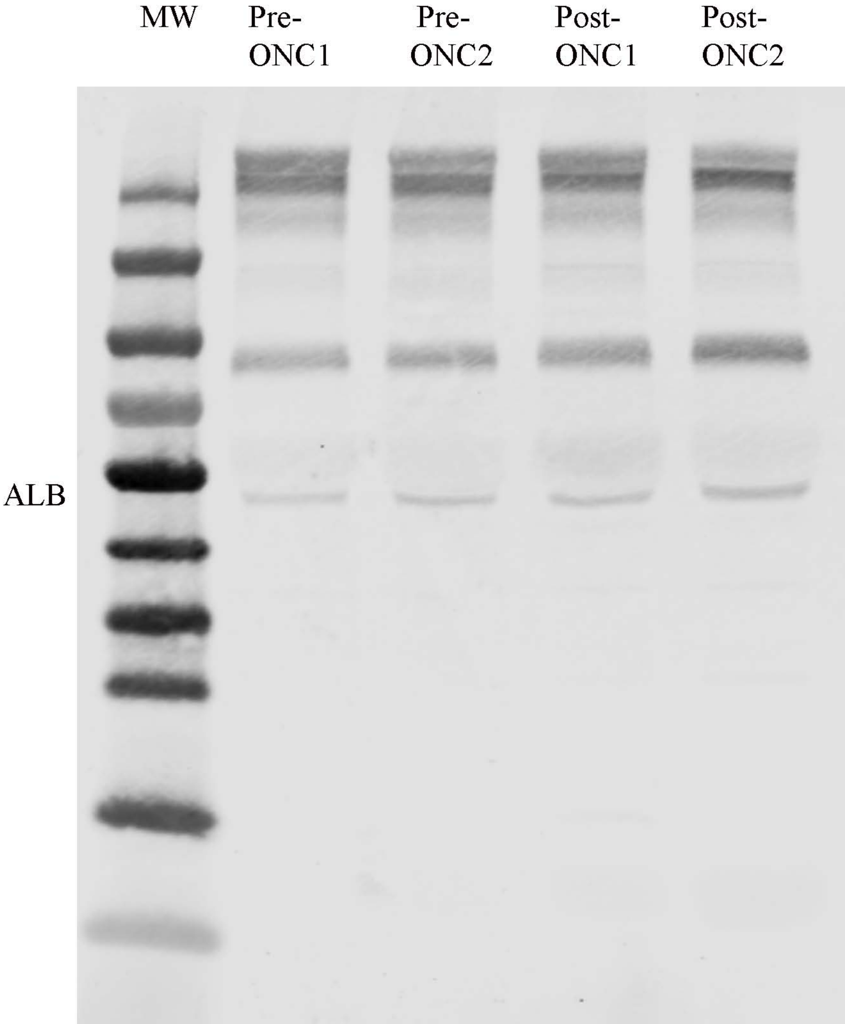

Fig. S20 - From Fig. 3B
Rho pulldown probing with
Rho antibody

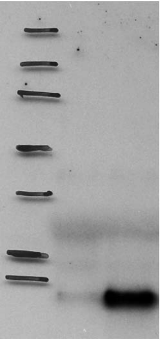

Activated
Rho

Con +Histone

Fig. S21 - From Fig. 3B, Total Rho from Lysates for normalization

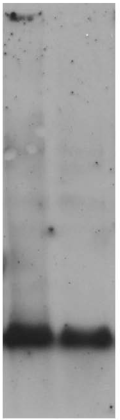

Fig. S22 from Fig 3C probing with p35 antibody

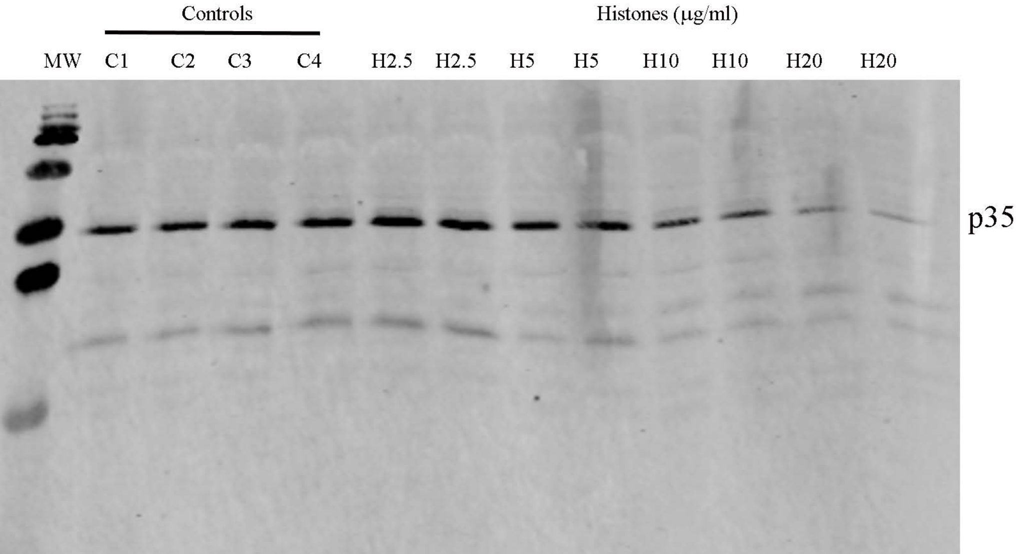

Fig. S23 from Fig. 3C probing with Actin antibody

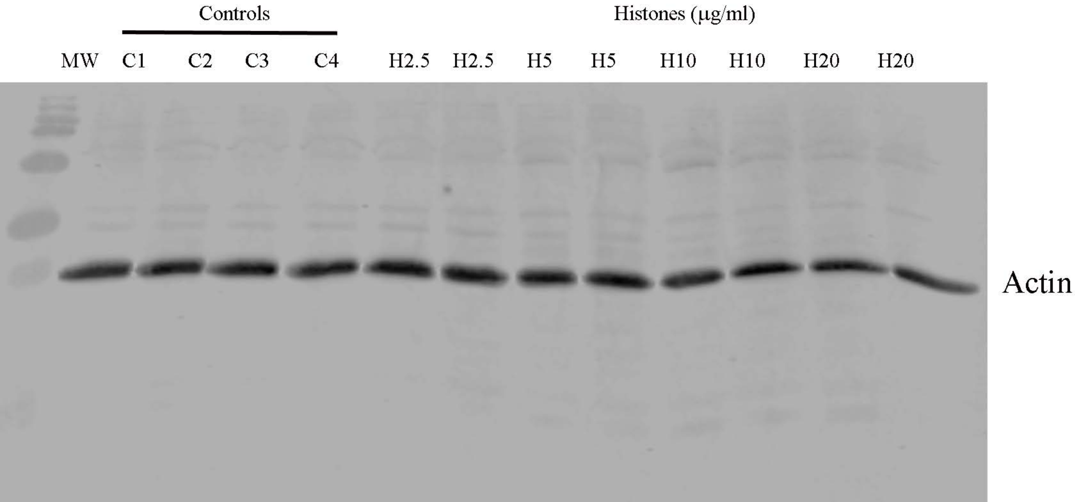

Fig. S24 from Fig. 8A probing with pYB-1 antibody

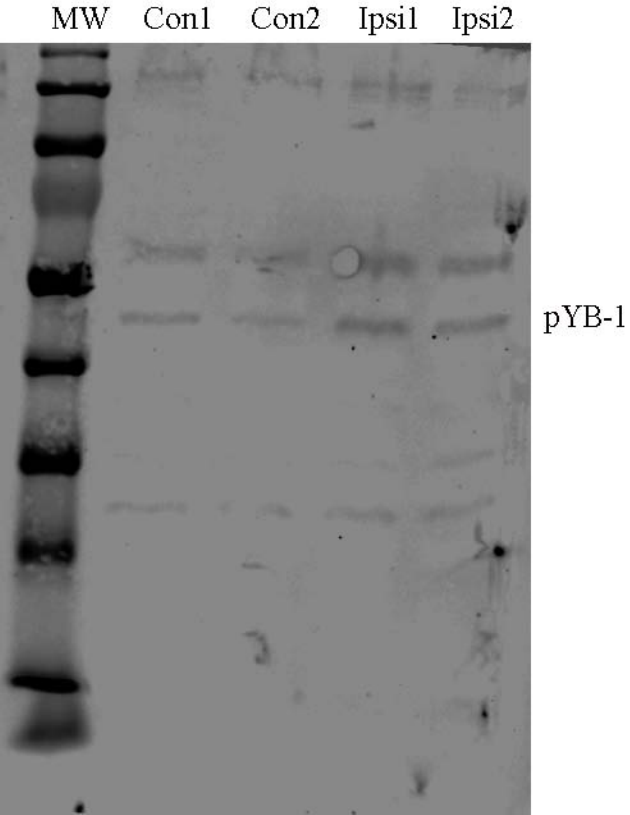

Fig. S25 from Fig. 8A probing for Total YB-1

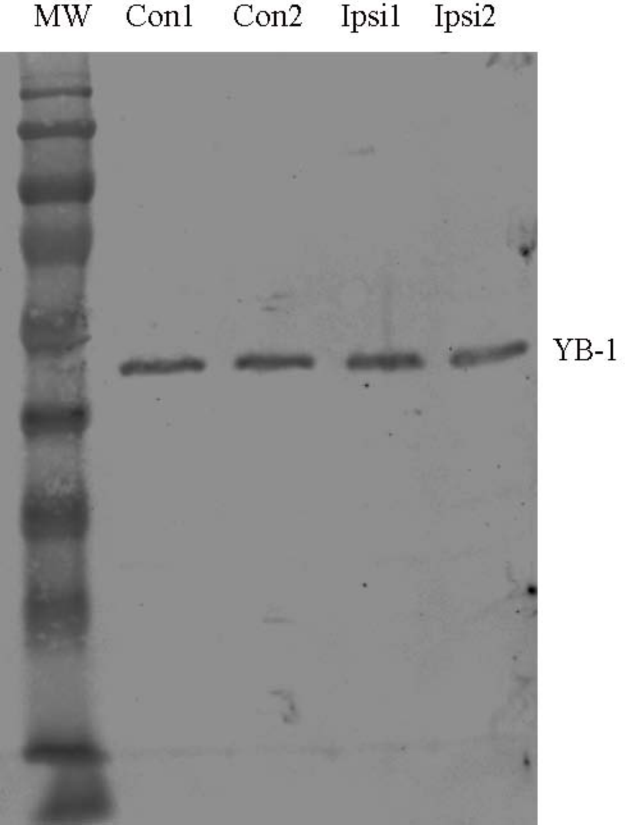

Fig. S26 from Fig. 8A probing for β-III Tubulin

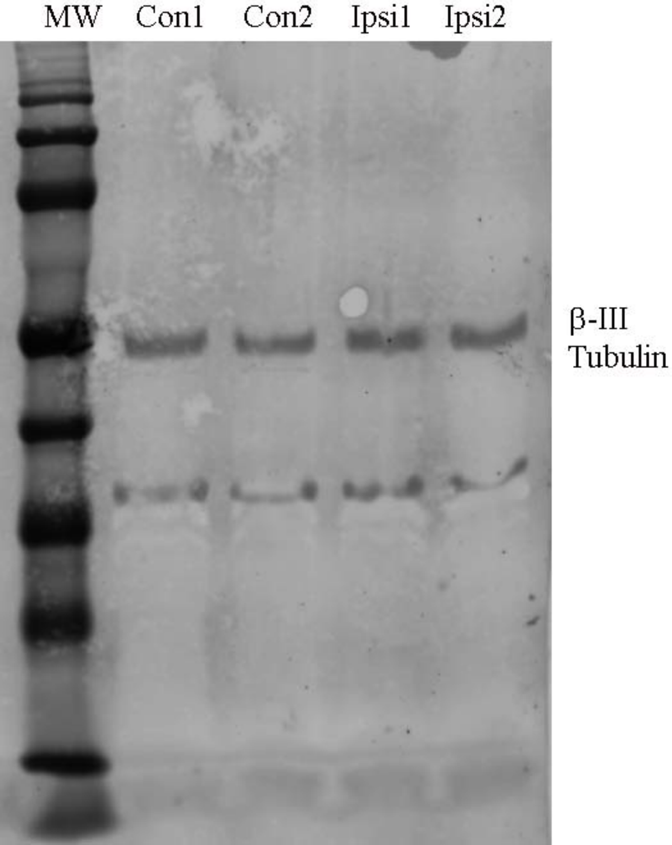

Fig. S27 from Fig. 8B probing with pYB-1 antibody

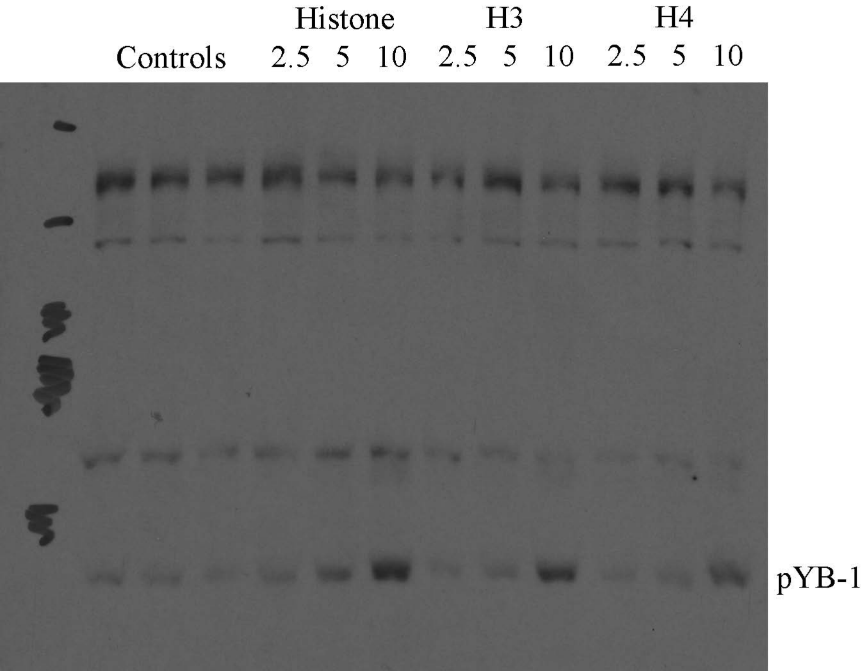

Fig. S28 from Fig. 8B probing for total YB-1

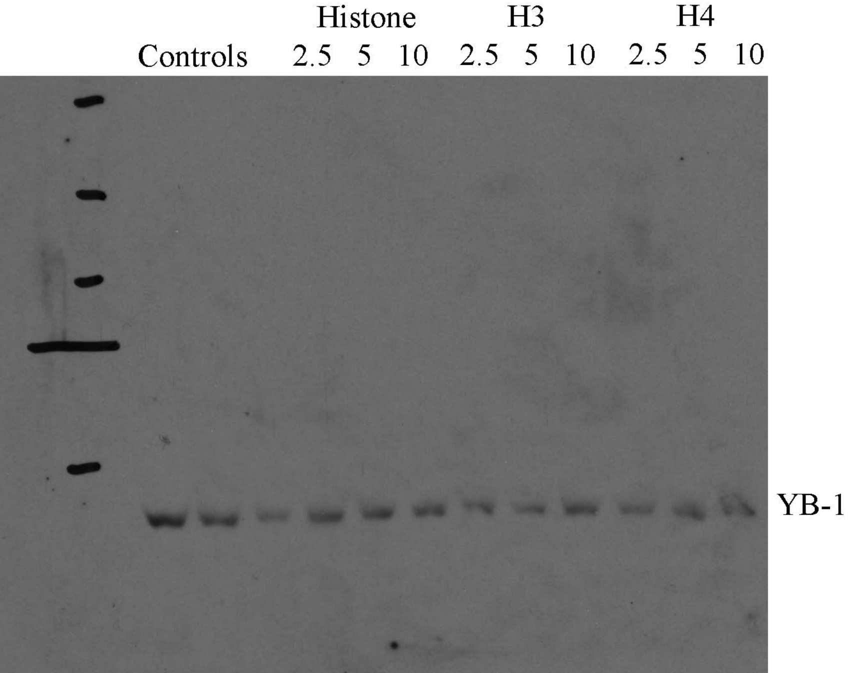

Fig. S29 from Fig. 8B probing with Actin Antibody

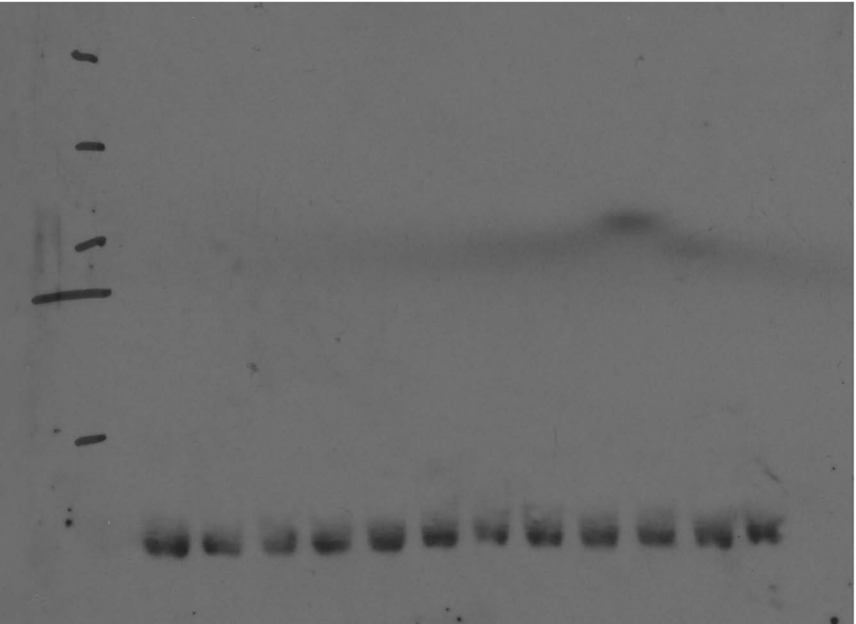

Fig. S30 from Fig. 8C probing with pYB-1 antibody

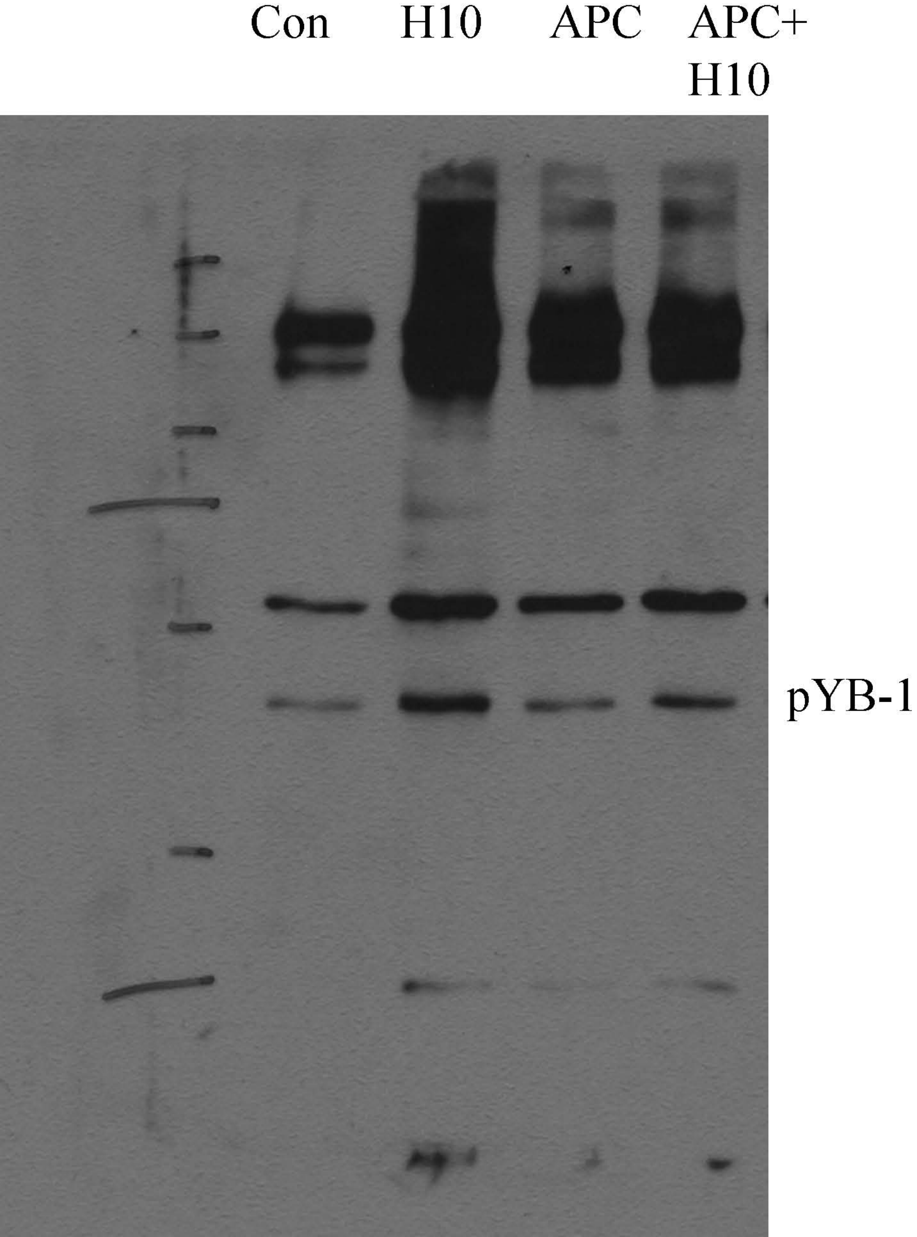

Fig. S31 from Fig. 8C probing with Actin Antibody

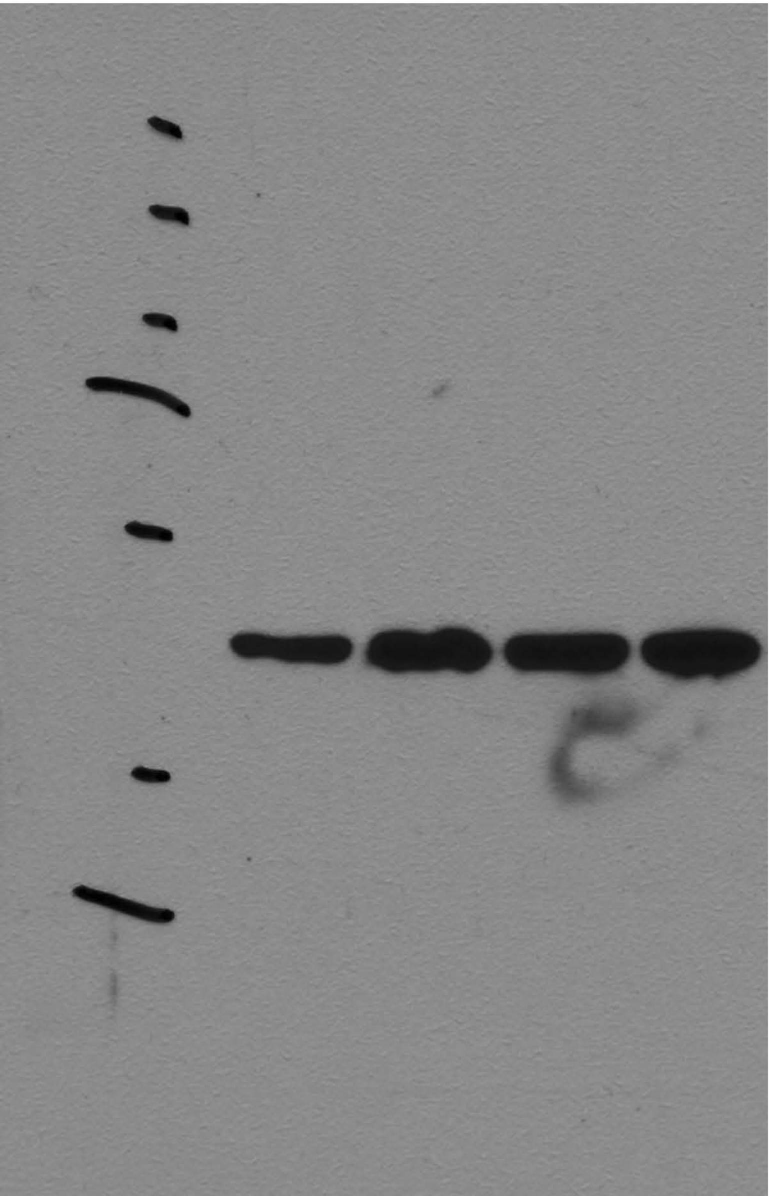

Fig. S32 from Fig. 8E probing with pYB-1 Antibody

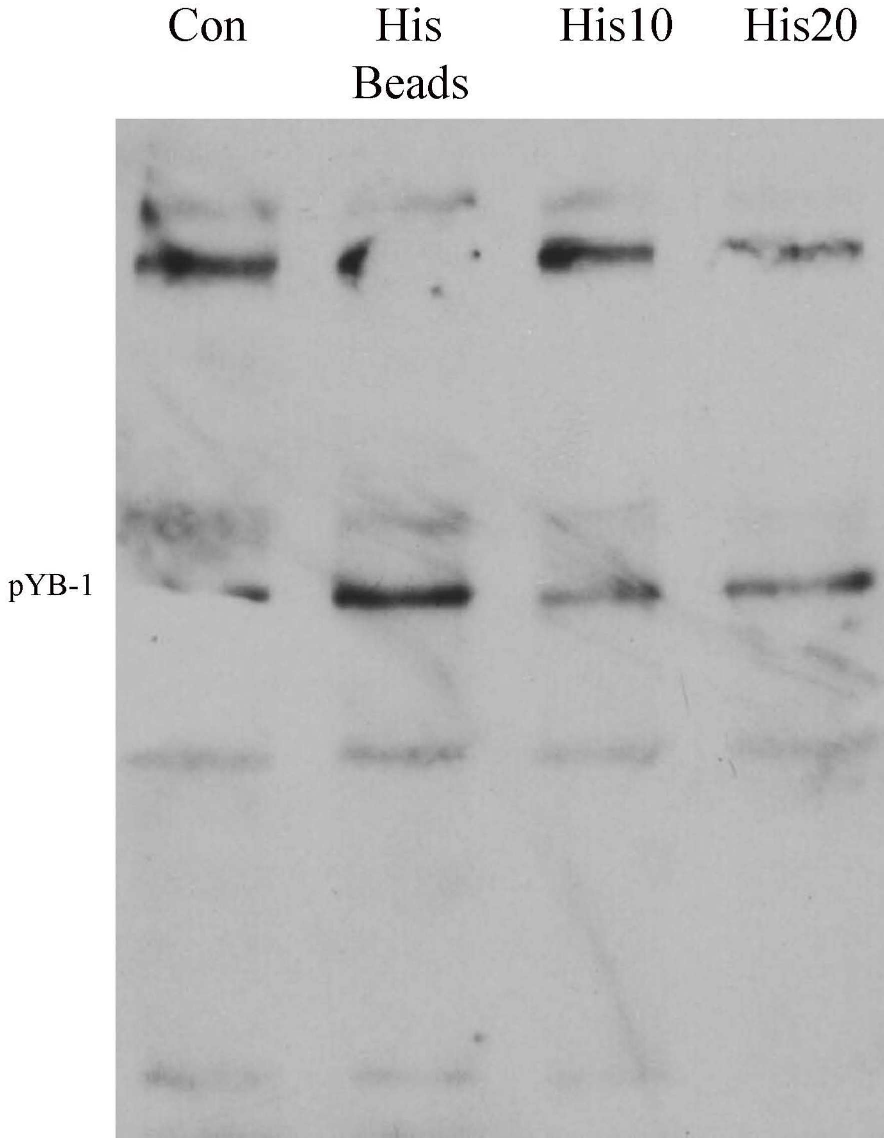

Fig. S33 from Fig. 8E probing with GAPDH Antibody

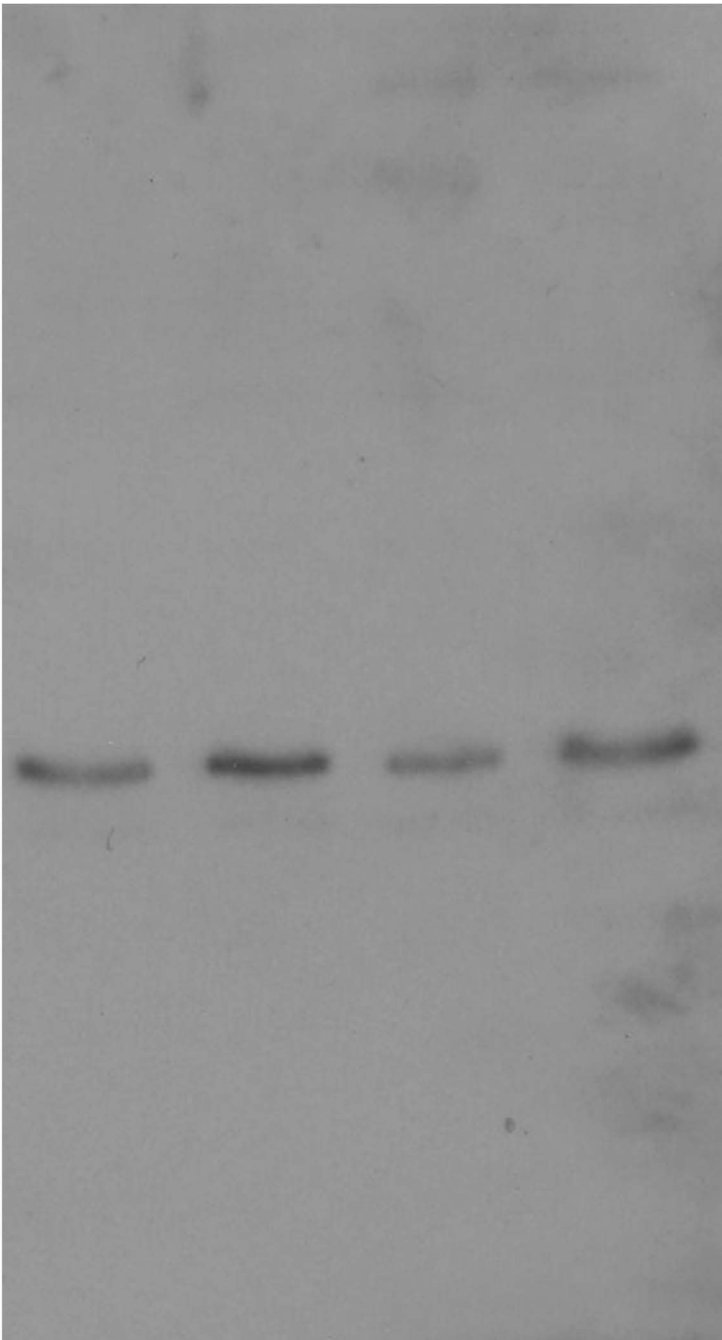

Supplement: fcab271_Supplementary_Data [file fcab271_Supplementary_Data.zip › Supplementary_Material.pdf]
